# Muscular Dystrophy-Associated *SUN1* and *SUN2* Variants Disrupt Nuclear-Cytoskeletal Connections and Myonuclear Organization

**DOI:** 10.1371/journal.pgen.1004605

**Published:** 2014-09-11

**Authors:** Peter Meinke, Elisabetta Mattioli, Farhana Haque, Susumu Antoku, Marta Columbaro, Kees R. Straatman, Howard J. Worman, Gregg G. Gundersen, Giovanna Lattanzi, Manfred Wehnert, Sue Shackleton

**Affiliations:** 1 Institute of Human Genetics and Interfaculty Institute of Genetics and Functional Genomics, University of Greifswald, Greifswald, Germany; 2 National Research Council of Italy - CNR - Institute for Molecular Genetics, Unit of Bologna IOR, Bologna, Italy; 3 Rizzoli Orthopaedic Institute, Laboratory of Musculoskeletal Cell Biology, Bologna, Italy; 4 Department of Biochemistry, University of Leicester, Leicester, United Kingdom; 5 Department of Pathology and Cell Biology, College of Physicians and Surgeons, Columbia University, New York, New York, United States of America; 6 Centre for Core Biotechnology Services, University of Leicester, Leicester, United Kingdom; 7 Department of Medicine, College of Physicians and Surgeons, Columbia University, New York, New York, United States of America; The Jackson Laboratory, United States of America

## Abstract

Proteins of the nuclear envelope (NE) are associated with a range of inherited disorders, most commonly involving muscular dystrophy and cardiomyopathy, as exemplified by Emery-Dreifuss muscular dystrophy (EDMD). EDMD is both genetically and phenotypically variable, and some evidence of modifier genes has been reported. Six genes have so far been linked to EDMD, four encoding proteins associated with the LINC complex that connects the nucleus to the cytoskeleton. However, 50% of patients have no identifiable mutations in these genes. Using a candidate approach, we have identified putative disease-causing variants in the *SUN1* and *SUN2* genes, also encoding LINC complex components, in patients with EDMD and related myopathies. Our data also suggest that *SUN1* and *SUN2* can act as disease modifier genes in individuals with co-segregating mutations in other EDMD genes. Five SUN1/SUN2 variants examined impaired rearward nuclear repositioning in fibroblasts, confirming defective LINC complex function in nuclear-cytoskeletal coupling. Furthermore, myotubes from a patient carrying compound heterozygous *SUN1* mutations displayed gross defects in myonuclear organization. This was accompanied by loss of recruitment of centrosomal marker, pericentrin, to the NE and impaired microtubule nucleation at the NE, events that are required for correct myonuclear arrangement. These defects were recapitulated in C2C12 myotubes expressing exogenous SUN1 variants, demonstrating a direct link between *SUN1* mutation and impairment of nuclear-microtubule coupling and myonuclear positioning. Our findings strongly support an important role for SUN1 and SUN2 in muscle disease pathogenesis and support the hypothesis that defects in the LINC complex contribute to disease pathology through disruption of nuclear-microtubule association, resulting in defective myonuclear positioning.

## Author Summary

Emery-Dreifuss muscular dystrophy (EDMD) is an inherited disorder involving muscle wasting and weakness, accompanied by cardiac defects. The disease is variable in its severity and also in its genetic cause. So far, 6 genes have been linked to EDMD, most encoding proteins that form a structural network that supports the nucleus of the cell and connects it to structural elements of the cytoplasm. This network is particularly important in muscle cells, providing resistance to mechanical strain. Weakening of this network is thought to contribute to development of muscle disease in these patients. Despite rigorous screening, at least 50% of patients with EDMD have no detectable mutation in the 6 known genes. We therefore undertook screening and identified mutations in two additional genes that encode other components of the nuclear structural network, *SUN1* and *SUN2*. Our findings add to the genetic complexity of this disease since some individuals carry mutations in more than one gene. We also show that the mutations disrupt connections between the nucleus and the structural elements of cytoplasm, leading to mis-positioning and clustering of nuclei in muscle cells. This nuclear mis-positioning is likely to be another factor contributing to pathogenesis of EDMD.

## Introduction

The nuclear envelope (NE) is composed of the nuclear membranes, nuclear lamina and nuclear pore complexes and encloses the chromatin in eukaryotic cells. Lamin intermediate filament proteins are the major structural components of the NE and polymerize to form a fibrous meshwork that underlies the nucleoplasmic face of the inner nuclear membrane. This nuclear lamina is attached to the inner nuclear membrane through interactions with multiple integral inner nuclear membrane (INM) proteins [1]. Together, these proteins form a structural network that plays a vital role in supporting the NE and maintaining nuclear integrity, whilst also contributing to chromatin organization and regulation of gene expression (reviewed in [2]).

Mutations in genes encoding NE proteins are associated with a range of tissue-restricted inherited disorders that can affect striated muscle, bone, fat or neurons and in some cases cause premature ageing syndromes [3]. Most strikingly, different mutations in one gene – the *LMNA* gene that encodes A-type nuclear lamins – can cause many diseases, which have collectively been termed laminopathies [4]. Diseases affecting striated muscle are the most common of the laminopathies and include autosomal dominant and recessive Emery-Dreifuss muscular dystrophy (EDMD2 and EDMD3, respectively; OMIM#181350), limb-girdle muscular dystrophy (LGMD) type 2B and dilated cardiomyopathy and conduction system disease (CMD) type 1A [5]–[8]. These diseases share the common feature of cardiomyopathy, but EDMD and LGMD also involve progressive muscle wasting and weakness. In all cases, premature sudden death can result from cardiac arrhythmia and conduction defects.

Striated muscle disease, in particular EDMD, can also be caused by mutations in genes encoding other NE proteins. An X-linked form of EDMD (EDMD1; OMIM#310300) is caused by mutations in *EMD*, that encodes the integral INM protein emerin [9]. Together, mutations in *LMNA* and *EMD* account for around 40% of cases of EDMD [10]. Rare mutations in the genes encoding FHL1, TMEM43 (also named LUMA), nesprin-1 and nesprin-2 have also been reported [11]–[13]. Interestingly, A-type lamins, nesprins and emerin all interact with each other [14]–[16], contributing to a network that connects the nuclear lamina to the cytoskeleton, termed the LINC (Linker of Nucleoskeleton and Cytoskeleton) complex [17]. Furthermore, interactions are often perturbed by muscle disease-causing mutations, indicating that this network of interactions plays an important role in muscle function [12], [18], [19].

The central components of the LINC complex in mammals are SUN and nesprin proteins that reside in the INM and outer nuclear membrane (ONM), respectively. The conserved SUN and KASH domains of the respective proteins interact in the perinuclear space to form a bridge spanning the INM, perinuclear space and ONM that connects the nuclear lamina to the cytoskeleton. The nucleoplasmic N-termini of the SUN proteins, SUN1 and SUN2, interact with the nuclear lamina, anchoring the LINC complex at the NE [20]–[22]. In turn, the cytoplasmic domains of the nesprins connect to the cytoskeleton. There are 4 nesprin isoforms encoded by different genes. Giant isoforms of nesprins-1 and -2 contain an N-terminal calponin homology domain responsible for actin binding [23], [24] and linkage to the centrosome through microtubules and their motor proteins [25]. Nesprin-3 connects to the cytoplasmic intermediate filament network through interaction with plectin [26], whilst nesprin-4 is specific to epithelial cells and connects the NE to microtubules via the kinesin-1 motor protein [27].

There are several proposed mechanisms to explain the tissue specificity of EDMD and other laminopathies, which centre around the “gene expression” and “structural” hypotheses [28]. Current evidence strongly supports the “structural hypothesis”, which suggests that muscle-associated laminopathies primarily result from weakening of the structural networks of the nuclear lamina and cytoskeleton and the LINC complex that connects these two networks [29]. Since myocytes are subject to recurrent mechanical strain from contractile forces, weakening of these structural networks renders the cells particularly susceptible to damage. However, the LINC complex is also vital for correct myonuclear positioning [30]–[33] and defects in this process are implicated in impaired muscle function [34], [35].

Despite the genetic studies so far carried out, causative mutations have been identified in only approximately 50% of EDMD and related muscle disease cases [10]. It is therefore highly likely that mutations in additional genes contribute to the disease. Furthermore, there is significant heterogeneity in disease severity even within families carrying the same gene mutation [36]–[40], which has led to the suggestion of modifier genes [41]–[43].

Given that SUN1 and SUN2 interact with at least four of the known muscle disease-associated NE proteins and that these interactions can be perturbed by disease-causing *LMNA* and *EMD* mutations [44], we investigated whether the *SUN1* and *SUN2* genes may also be mutated in some individuals. Screening of the *SUN1* and *SUN2* genes in a large cohort of patients with EDMD and phenotypically related myopathies identified *SUN1* and/or *SUN2* variants in several patients. Presence of *SUN1* or *SUN2* variants correlated with increased disease severity in patients with EDMD carrying mutations in other genes, thus identifying *SUN1* and *SUN2* as modifiers of the EDMD disease phenotype. We further provide evidence that these mutations disrupt nuclear-cytoskeletal connection and nuclear positioning, supporting the hypothesis that muscular dystrophies arise from defective nuclear-cytoskeletal coupling.

## Results

### Screening *SUN1* and *SUN2* genes in a cohort of patients with EDMD and related myopathies

We analyzed DNA from 175 unrelated patients with EDMD and related myopathies, who had previously undergone screening for mutations in the *LMNA*, *EMD*, *SYNE1/SYNE2 alpha* and *beta* (encoding short isoforms of nesprin-1 and nesprin-2, respectively) and *FHL1* genes and in whom no causative mutation had been found. These included both sporadic cases and index patients from familial cases. Furthermore, there have been several reports of modifiers of the phenotype of *LMNA*-linked muscle diseases [41]–[43]. We therefore also screened EDMD patients carrying identified *LMNA*, *SYNE1/SYNE2 alpha and beta* and *EMD* mutations to determine whether mutation of *SUN1* or *SUN2* may influence disease phenotype. Most individuals were of Caucasian origin, except where otherwise stated.

**Figure 1:**
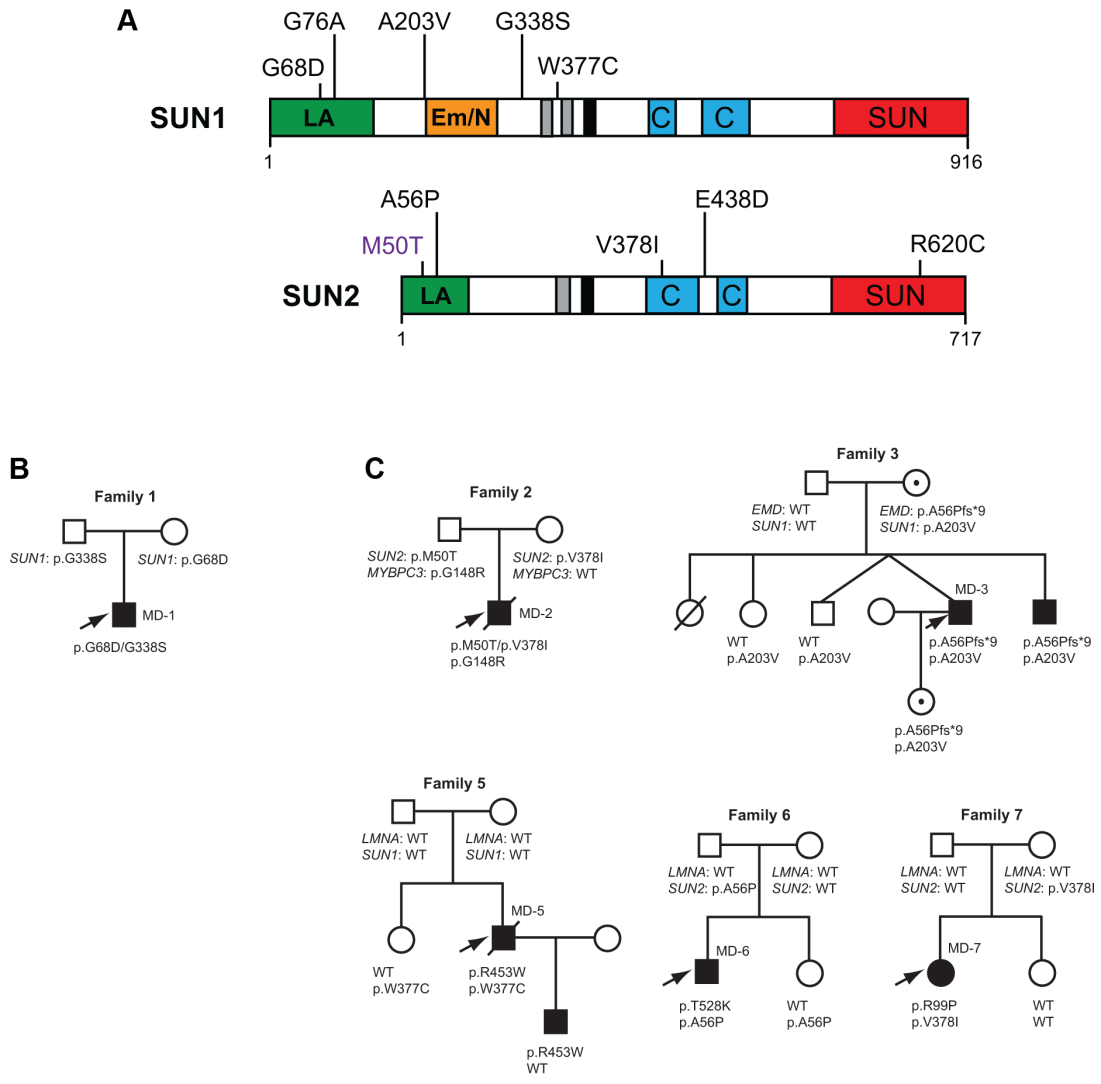
SUN1 and SUN2 variants identified and associated family pedigrees. (**A**) Schematic diagram of the SUN1 and SUN2 protein domain organization and locations of disease-associated variants identified in our cohort. Mutation *SUN1* M50T, indicated in purple, did not disrupt LINC complex function in migration assays and thus may not be truly disease-causing. The mapped lamin A/C (green) and emerin (orange) binding sites, located in the nucleoplasmic N-terminal domain, are indicated. Regions of high hydrophobicity and the transmembrane domain are shown in grey and black, respectively. Coiled-coil domains responsible for oligomerization (blue) and the highly conserved SUN domain (red), found within the luminal C-terminal domain, are also indicated. (**B**) Pedigree with recessive inheritance of compound heterozygous *SUN1* variants. (**C**) Pedigrees where severely affected index cases carry *SUN1* and/or *SUN2* variants in combination with other gene mutations. Filled circles/squares indicate affected females/males. Circles containing a dot, in family 3, indicate unaffected female carriers of the X-linked *EMD* mutation. Arrows indicate index patients.

The 23 exons of the *SUN1* gene (see Figure S1) and 19 exons of the *SUN2* gene (ENSG00000100242, ENST00000405510), including intron/exon boundaries and promoter regions were analyzed. DNA was amplified using PCR and analyzed by direct Sanger sequencing. In total, we found 34 single nucleotide polymorphisms within the coding regions of *SUN1* and *SUN2*, 18 of which were classified as rare, non-synonymous changes following analysis of their frequencies in sequenced genome databases (Table S1, Figure S3). Three of these variants did not segregate with disease in the respective families (Figure S2). In nine unrelated families or sporadic cases, however, we identified 10 rare non-synonymous variants in *SUN1* and *SUN2* for which we have obtained evidence of pathogenic effects, as deduced from genetic, phenotypic and/or functional data (Figure 1A).

### Putative disease-associated *SUN1* and *SUN2* variants in patients with EDMD-like phenotypes

We identified 5 rare, non-synonymous *SUN1* and/or *SUN2* variants in 3 individuals who lacked mutations in other genes but had EDMD or related myopathy phenotypes (Table 1). Sporadic patient MD-11 carried a single *SUN2* p.R620C sequence variation. We had no access to DNA from family members for this patient, but the high degree of evolutionary conservation of R620 is supportive of disease-association (Figure S3). Patient MD-1 carried compound heterozygous *SUN1* p.G68D and p.G338S variants. For patient MD-1 we had access to DNA from family members and observed apparent recessive inheritance, with one mutation coming from each of the unaffected parents (Figure 1B). *SUN1* p.G338S was also present in the reference population at low frequency (see Table S1). These residues are located within the poorly conserved N-terminal domain of the protein and, in this context, are moderately conserved (Figure S3), but functional data presented below present compelling evidence of the involvement of both mutations in disease causation. Sporadic patient MD-12 carried heterozygous changes in both *SUN1* and *SUN2*, encoding SUN1 p.W377C and SUN2 p.E438D, respectively. E438 is conserved in mammals, whilst W377 is conserved across all species examined (Figure S3).

**Table 1:** Putative disease-causing variants in *SUN1* and *SUN2* in patients with EDMD-like phenotypes.

Family	Index case	SUN1 variant	SUN2 variant	Other mutations	Disease phenotype
1	MD-1	p.G68D p.G338S	none	none	Male; age at onset 10 years; mild muscle weakness; rigid spine; serum creatine kinase elevation 6X; no cardiac involvement; last clinical examination at age 10 years; sporadic case.
11	MD-11	none	p.R620C	none	Sporadic EDMD-related myopathy, no other clinical information available.
12	MD-12	p.W377C	p.E438D	none	Heart rhythm disturbances at age 34 years; partial lipodystrophy on left lower leg; sporadic case

### *SUN1* and *SUN2* variants with disease modifying effects in patients with co-segregating mutations in other genes

Because patients with EDMD-like phenotypes exhibit variable disease severity that could be explained by mutations or polymorphisms in additional genes, we screened for *SUN1* and *SUN2* variants in patients with known mutations in causative genes. *SUN1* or *SUN2* variants were indeed present in some patients from families with *LMNA* or X-linked *EMD* mutations (Table 2; Figure 1C). These sequence changes correlated with increased disease severity. In one example, a *SUN1* p.A203V polymorphism in patient MD-3 co-segregated with a previously reported *EMD* p.L84Pfs*6 mutation in two brothers with unusually severe EDMD (Figure 1C, Family 3) [39]. The *EMD* p.L84Pfs*6 mutation, which abolishes emerin expression, has been reported in an unrelated family, where the course of the disease was significantly milder EDMD with later age of onset and no loss of ambulation [45]. Another unrelated patient carrying *EMD* p.L84Pfs*6 was included in this study but no *SUN1* or *SUN2* variants were found and their phenotype was similar to that described by Manilal et al. [45]. In another case, the *SUN1* p.G76A mutation, when combined with *EMD* p.A56Pfs*9 in a previously described Korean patient (MD-4), led to a very severe clinical picture with complete atrioventricular block requiring pace maker implantation at age 14 years [46]. Similarly, *SUN1* p.W377C was detected in combination with *LMNA* p.R453W in patient MD-5. This individual had severe disease and died early at the age of 34 years from heart failure. The patient's son, carrying *LMNA* p.R453W only, did not show clinical signs of contractures or muscular weakness at age 10 years. *LMNA* p.R453W is a common EDMD-associated *LMNA* mutation and is generally not associated with severe cardiac disease, suggesting that, in patient MD-5, *SUN1* p.W377C had a modifying effect to increase disease severity [47], [48]. The same SUN1 p.W377C variant was detected in patient MD-12, who had an EDMD-like phenotype but did not have mutations in *EMD* or *LMNA* but carried a concurrent *SUN2* p.E438D variant, as described above.

**Table 2:** *SUN1* and *SUN2* variants with disease-modifying effects in patients with *MYBPC3*, *EMD* and *LMNA* mutations.

Family	Index case	SUN1 variant	SUN2 variant	Other mutations	Disease phenotype
2	MD-2	none	p.M50T p.V378I	*MYBPC3* p.G148R	Male; age at onset 6 months; hypertrophic cardiomyopathy; at age 9 years ECG showed cardiac arrhythmia, supraventricular extrasystols, Echocardiogram: right ventricular septum hypertrophy, first degree atrioventricular block; no muscular weakness or dystrophy; sinus tachycardia and bradycardia, mild left ventricular functional impairment; died at age16 years from heart failure; sporadic case.
3	MD-3	p.A203V	none	*EMD* p.L84Pfs*6	Male; age at onset 2 years; severe contractures of neck, thoracolumbar spine, elbows, and Achilles tendons; Achillotomia at age 6; loss of ambulation at age 15; moderate to severe muscle weakness; left anterior hemi-block and ventricular ectopy at age 23; ventricular dilation at age 33; X-linked EDMD [39].
4	MD-4	p.G76A	none	*EMD* p.A56Pfs*9	Male; age of onset 1 year, wasting and weakness of shoulder girdle and limb-girdle muscles; at age 14 severe contractures of neck, elbow and Achilles tendons, tendon reflexes absent; at age 14 dilated right atrial and ventricular dilation, atrial fibrillation, complete AV block and junctional escape rhythm; pacemaker since age 14; CK elevation 4X; X-linked EDMD [46].
5	MD-5	p.W377C	none	*LMNA* p.R453W	Male; age of onset 8 years; slowly progressive humero-peroneal muscular weakness; since age 14 rigid spine, contractures of elbow and Achilles-tendons; at age 25 cardiac disturbances; AV-block III; heart pacemaker at age 31; died at age 34 of heart failure; autosomal dominant EDMD.
6	MD-6	none	p.A56P	*LMNA* p.T528K	Male; age at onset 1 year; delayed early childhood developmental mile stones; later difficulties in climbing stairs, muscular weakness; at age 15 tachycardia, extrasystols; at age 17 intra-ventricular cardiac conduction defects, contractures of elbow and Achilles tendons; proximal humero-peroneal muscle atrophy, rigid spine, Gower's maneuver; CK elevated 3–5X; *de novo LMNA* mutation leading to sporadic EDMD.
7	MD-7	none	p.V378I	*LMNA* p.R99P	Female; age of onset 4 years, diffuse muscular weakness; later bilateral contractures of the elbows and ankles, dilated cardiomyopathy, first degree atrioventricular block; at age 14 heart pacemaker; histopathology showed fibrosis of the heart muscle; at age 15 heart transplantation; *de novo LMNA* mutation leading to sporadic EDMD.

We detected *SUN2* mutations in combination with *LMNA* mutations in two additional index cases. Patient MD-6 carried *LMNA* p.T528K and *SUN2* p.A56P, whilst patient MD-7 carried *LMNA* p.R98P and *SUN2* p.V378I (Table 2; Figure 1C). In both cases, the *LMNA* mutation had arisen *de novo*, whilst the *SUN2* mutation was inherited from an unaffected parent. Again, the disease expression in both index patients was more severe than is typical for EDMD [49], [50], with early onset at age 1 and 4 years, respectively, and early heart involvement including heart transplantation before age 20 years (Table 2).

We also identified two *SUN2* variants, encoding variants p.M50T and pV378I, in patient MD-2 who had hypertrophic cardiomyopathy and also carried a mutation in *MYBPC3* (p.G148R), which encodes a myosin binding protein. The same *MYBPC3* mutation was previously reported in a Dutch family, where a severely affected index patient had compound heterozygous mutations in *MYCBP3* but other family members carrying only p.G148R were either asymptomatic or developed cardiomyopathy late in life [51]. This *MYBPC3* mutation was present in both patient MD-2 and his father (Figure 1C; S. Waldmueller, personal communication). Patient MD-2 presented as a 6 month-old boy with hypertrophic cardiomyopathy and died at 16 years from heart failure. His father, who carries the *SUN2* p.M50T variant but not p.V378I, is asymptomatic. Thus, the *SUN2* p.V378I variant appears to have a dramatic effect on disease severity. Notably, this variant is present in the reference population at low frequency (see Table S1), suggesting that it may be a relatively common genetic modifier of inherited cardiomyopathy.

### SUN1 and SUN2 disease-associated variants disrupt centrosome reorientation and nuclear movement in NIH3T3 fibroblasts

Our genetic results suggest that mutations or polymorphisms in *SUN1* and *SUN2* may cause muscular dystrophy and act as modifiers of EDMD and cardiomyopathy. To obtain additional evidence that these variants play a role in pathophysiology, we examined the effects of several variants on a known function of the LINC complex, namely centrosome orientation and nuclear movement in migrating cells. SUN2, along with the nesprin-2G isoform, assembles into transmembrane actin-associated nuclear (TAN) lines that couple actin cables to the nucleus to move it rearward and reorient the centrosome toward the leading edge in migrating NIH3T3 fibroblasts [52], [53]. While SUN1 is not in TAN lines, it also functions in connecting the nucleus to the cytoskeleton via the LINC complex.

**Figure 2:**
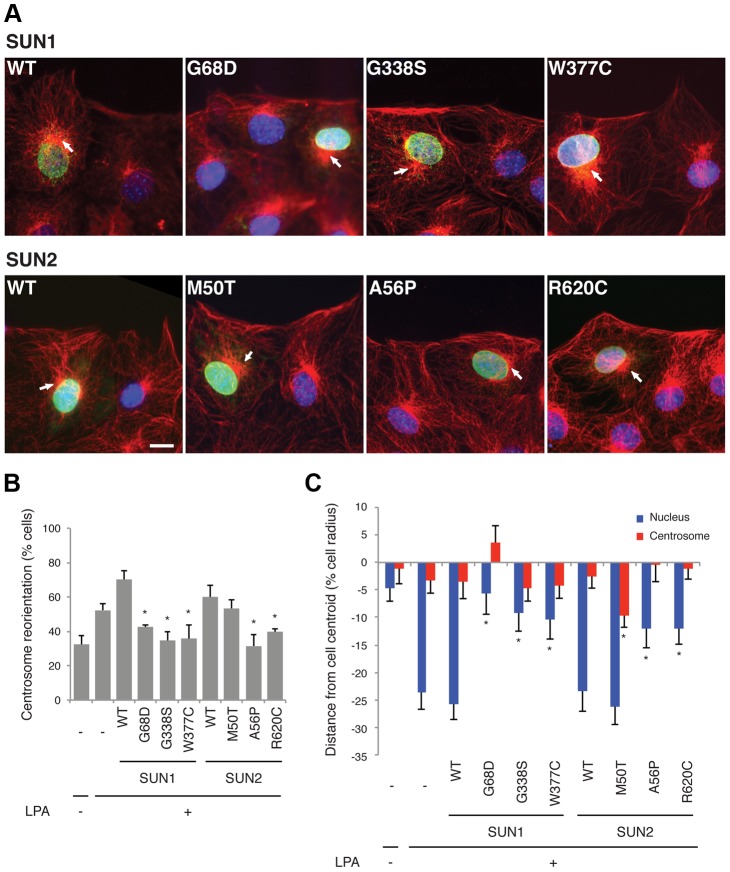
SUN1 and SUN2 variants disrupt nuclear-cytoskeletal coupling in NIH3T3 fibroblasts. (**A**) Representative immunofluorescence micrographs of LPA-stimulated NIH3T3 fibroblasts expressing SUN1 or SUN2 wild-type (WT) or variant proteins. Cells were immunostained for myc (green), tubulin (red) and DAPI (blue). Location of centrosome (arrows) was determined by the center of microtubule array. Bar, 20 µm. (**B**) Quantification of centrosome reorientation in LPA-stimulated NIH3T3 fibroblasts expressing the indicated SUN variants. Significant differences are indicated by * with α<0.05 based on Student's t-test when the sample is compared to non-expressing NIH3T3 fibroblasts. (**C**) Quantification of nucleus and centrosome position relative to the cell centroid in NIH3T3 fibroblasts expressing the indicated SUN variants. Positive values are toward the leading edge, negative values toward the cell rear. Data are from at least three independent experiments for each sample. Significant differences are indicated by * with α<0.05 based on Student's t-test when the sample is compared to non-expressing NIH3T3 fibroblasts.

We expressed three myc-SUN1 and three myc-SUN2 variants in NIH3T3 fibroblasts at the edge of a wounded monolayer by DNA microinjection and stimulated nuclear movement and centrosome reorientation with the serum factor, lysophosphatidic acid (LPA). Upon stimulation, non-expressing NIH3T3 cells or NIH3T3 cells exogenously expressing wild-type (WT) SUN1 or SUN2, as well as the variant SUN2 M50T, reoriented their centrosomes (Figure 2A–B). Notably, *SUN2* p.M50T did not appear to influence disease severity in family 2. In contrast, cells expressing the putative disease-causing SUN1 variants, G68D, G338S or W377C, inhibited centrosome reorientation by blocking rearward positioning of the nucleus (Figure 2A–B). Similarly, cells expressing SUN2 A56P or R620C failed to reorient their centrosome due to an inability to position their nuclei rearward of the cell centroid (Figure 2A–B). All of the expressed SUN1 and SUN2 variants had a normal nuclear localization similar to the wild-type proteins (Figure 2A). The centrosome orientation defect in cells expressing the SUN variants occurred due to defective rearward nuclear movement and not disruption of positioning of the centrosome at the cell centroid (Figure 2C). Hence, five putative disease-causing or disease-modifying SUN variants blocked rearward nuclear movement in migrating NIH3T3 fibroblasts and it can be concluded that these mutants disrupt LINC complex function.

### Expression of LINC complex components is altered in patient myoblasts with compound heterozygous *SUN1* mutations

**Figure 3:**
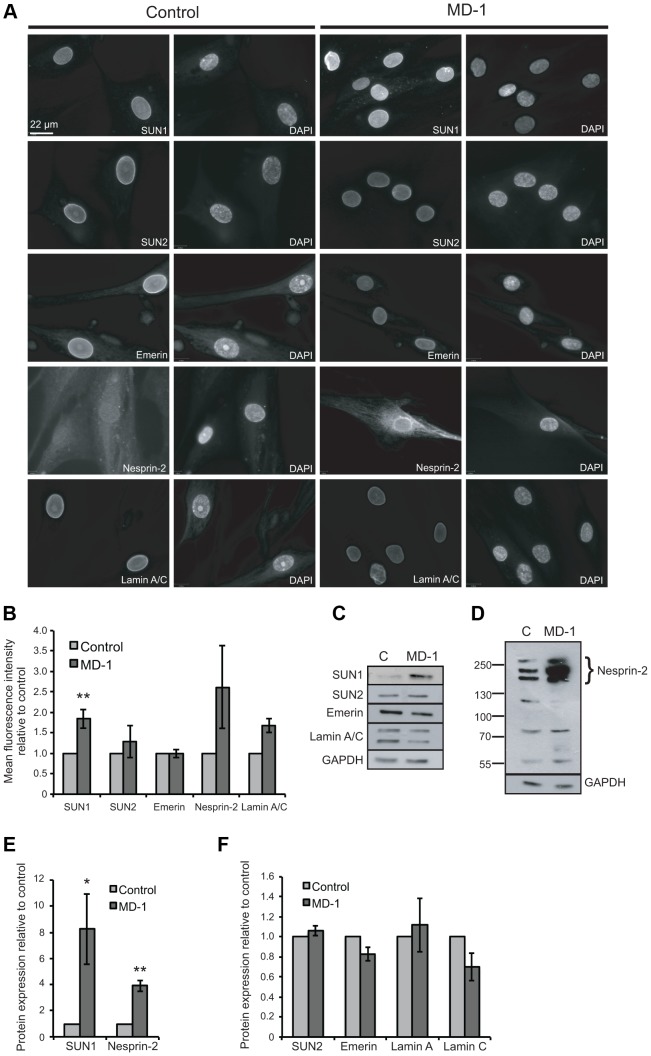
Expression of LINC complex proteins is increased in patient MD-1 (*SUN1* p.G68D/p.G338S) myoblasts. (**A**) Control and MD-1 myoblasts were fixed in methanol and analysed by immunofluorescence microscopy using SUN1, SUN2, emerin, nesprin-2G and lamin A/C antibodies, as indicated, together with DAPI staining of DNA. Scale bar, 22 µm. (**B**) Mean fluorescence intensity of SUN1, SUN2, emerin, nesprin 2 and lamin A/C was measured in individual DAPI-stained nuclei using an Olympus Scan∧R screening station and analysed using Scan∧R analysis software. The results are presented as mean ± S.E. of 1000 cells taken from at least 3 independent experiments. **P≤0.05. Significant *P*-value for SUN1 was *P* = 0.009. (**C**) Total protein extracts from control (C) and patient MD-1 myoblasts were Western blotted using antibodies against LINC complex-associated proteins, as indicated. (**D**) Samples prepared as in A were Western blotted using nesprin-2 (N2–N3) antibodies. (**E–F**) Protein expression was quantified by densitometric analysis of at least 3 independent experiments. The results are presented as mean ± S.E. *P≤0.05 and **P≤0.01. Each significant *P*- values are as follows: SUN1 *P* = 0.05, α-tubulin *P* = 0.003, nesprin-2 *P* = 0.002. *P*- value for emerin was *P* = 0.06.

Having established that some of the variants identified in our patient cohort disrupted LINC complex function in fibroblasts, we next wished to examine their role in muscle cells. We were able to obtain primary myoblasts from patient MD-1, carrying compound heterozygous *SUN1* p.G68D/p.G338S variants. To gain initial insight into the cellular effects of these mutations, we examined expression of LINC complex components and known SUN1 binding partners in the myoblasts by immunofluorescence microscopy. Since nesprin-1 is not significantly expressed in myoblasts [54], [55] (Figure S4), we stained for nesprin-2, lamin A/C, emerin, SUN1 and SUN2. No obvious defects in localization of SUN1, SUN2 or their interacting NE partners were observed, but expression of SUN1 and nesprin-2 at the NE was enhanced in the patient myoblasts (Figure 3A–B). Quantification of fluorescence intensity suggested that their expression was increased approximately 2-fold.

Since fluorescence intensity does not always provide an accurate reflection of total protein levels, we then examined total protein expression level by Western blot and found that SUN1 levels were elevated 8-fold in the patient versus control myoblasts (Figure 3C,E). Consistent with the fact that SUN proteins form complexes with, and are responsible for anchoring of nesprins in the ONM, we also observed a 4-fold increase in expression of the intermediate-sized muscle-enriched isoforms of nesprin-2 [55] in the patient myoblasts (Figure 3D–E). However, it remains possible that the bands observed are degradation products of nesprin-2 giant. In contrast, levels of SUN2, lamin A/C and emerin were not significantly altered in the patient cells (Figure 3C,F). To exclude the possibility that the observed changes were due to different levels of background differentiation in the control and patient cultures, we quantified expression of myogenin, an early marker of myogenic differentiation, and found no detectable level in either culture (data not shown).

To address the mechanism of SUN1 elevation in the patient myoblasts, we examined *SUN1* mRNA levels by qPCR and found no significant increase in mRNA level compared to the control, indicating that the p.G68D/p.G338S *SUN1* variants do not lead to increased mRNA levels (Figure S4). However, in analyzing mRNA levels of other LINC complex-associated proteins, we observed a statistically significant increase in expression of *LMNA*, *SUN2*, *SYNE1* and *SYNE2*. In contrast, *EMD* (encoding emerin) expression was decreased in the patient relative to control myoblasts (Figure S4).

### SUN1 interaction with emerin is impaired in MD-1 myoblasts

**Figure 4:**
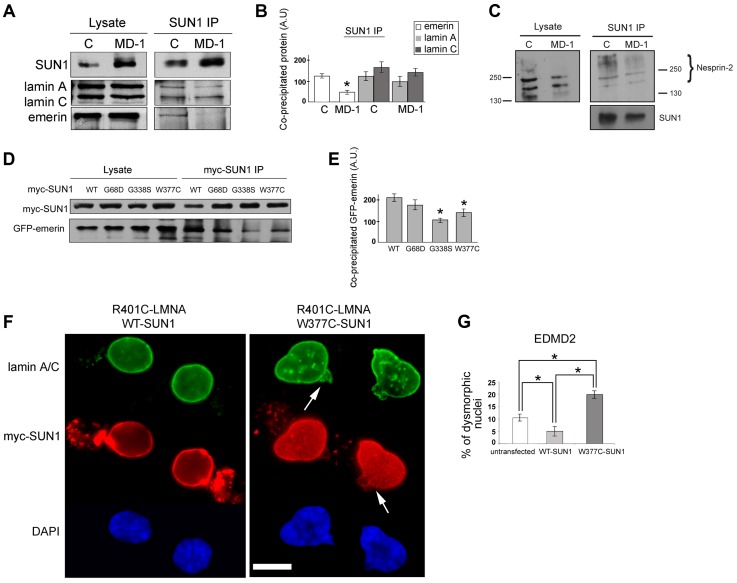
Emerin binding to p.G68D/p.G338S SUN1 is reduced *in vivo*. (**A**) SUN1 was immunoprecipitated from control (C) or MD-1 myoblast soluble lysates using 2383 SUN1 antibodies and samples Western blotted to detect co-immunopreciptated proteins. (**B**) Densitometric analysis of SUN1, lamin A/C and emerin bands from immunoprecipitated samples is plotted in arbitrary units (A.U.). (**C**) SUN1 was immunoprecipitated from control or MD-1 myoblasts and co-precipitated nesprin-2 was detected using N2–N3 antibody. Size markers (kDa) are indicated. Note that larger nesprin-2 isoforms are enriched in the immunoprecipitate compared to the lysate. (**D**) HEK293 cells were co-transfected with myc-SUN1 mutant and GFP-emerin plasmids and harvested after 48 hours. Co-precipitated GFP-emerin was detected by immunoblotting using GFP antibodies (myc-SUN1 IP). (**E**) Densitometric values of immunoblotted GFP-emerin bands are reported in arbitrary units (A.U.). (**F**) Human fibroblasts from an EDMD2 patient carrying the R401C *LMNA* mutation were transfected with SUN1-WT or SUN1-W377C cDNAs and fixed 48 hours after transfection. Lamin A/C was labelled using specific antibodies and revealed by FITC-conjugated secondary antibody (green). SUN1 was detected using Cy3-conjugated anti-myc antibody (red). Nuclei were counterstained with DAPI. Images show pairs of daughter cells from recent cell divisions. Increased dysmorphic nuclei with nuclear blebbing and honeycomb structures (arrows) are observed in double-mutant cells. The distance between daughter cells has been reduced using Photoshop 7 to allow magnification. (**G**) Percentage of dysmorphic nuclei in EDMD2 cells left untreated (untransfected), transfected with WT-SUN1 or transfected with W377C-SUN1 is reported in the graph as the mean of three independent experiments. Statistically significant differences (P<0.05) relative to controls or WT-transfected samples are indicated by asterisks.

One hypothesis to explain NE-associated muscle diseases is the “structural hypothesis”, which suggests that muscle damage occurs due to weakening of the protein interaction network supporting the NE [28], [56]. To determine whether muscle disease-associated SUN1 alterations disrupt interactions with other LINC complex components, we performed immunoprecipitation with anti-SUN1 antibodies on protein extracts from control and patient MD-1 myoblasts and detected co-precipitating proteins. We observed a reproducible reduction in SUN1 interaction with emerin in the patient myoblasts, whilst interaction with lamin A/C was not obviously perturbed (Figure 4A–B). Consistent with the enhanced recruitment of nesprin-2 to the NE, interaction of SUN1 with nesprin-2 was also maintained in the MD-1 myoblasts (Figure 4C). Larger isoforms of nesprin-2 were enriched in the immunoprecipitate compared to the initial lysates, indicating a preferential interaction of SUN1 with these less abundant isoforms. Thus, the SUN1 p.G68D/p.G338S variants in patient MD-1 appear to specifically disrupt interaction with emerin.

We confirmed defective interactions with emerin in HEK293 cells transiently expressing GFP-emerin and myc-SUN1 constructs. SUN1 was immunoprecipitated using anti-myc antibodies and co-precipitating GFP-emerin detected by Western blotting. We observed a significant reduction in interaction of emerin with both SUN1 G338S and W377C, whilst there was only a modest decrease in interaction with SUN1 G68D (Figure 4D–E). This correlates with the close proximity of G338 and W377 to the emerin binding site on SUN1 (see Figure 1A; [44]).

### SUN1 W377C increases the severity of nuclear defects in EDMD2 fibroblasts

Abnormalities in nuclear morphology and a honeycombed pattern of protein expression at the NE are commonly observed in EDMD fibroblasts obtained from patients with *LMNA* or *EMD* mutations [57]–[59]. We did not observe any obvious defects in nuclear morphology or protein localization in myoblasts from patient MD-1 (Figure 3A) or in cells transiently expressing a range of SUN1 or SUN2 mutants (for example, see Figure 2). However, we sought to determine whether SUN1 mutants have a modifying effect to increase the severity of nuclear defects when expressed in combination with mutations in *LMNA* or *EMD*. To achieve this, we expressed SUN1 W377C, found in combination with a *LMNA* R453W mutation in patient MD-5, in fibroblasts obtained from an EDMD2 patient carrying *LMNA* R401C. Cells expressing SUN1 W377C had a 4-fold increase in the level of nuclear dysmorphology, in terms of increase in nuclear blebbing and formation of honeycomb structures (Figure 4F, arrows), compared to those expressing WT SUN1 (Figure 4F–G). Cells expressing WT SUN1 had a 2-fold reduction in nuclear abnormalities compared to untransfected cells, suggesting a protective effect of SUN1 over-expression.

### MD-1 myoblasts exhibit an enhanced rate of differentiation but myonuclei are disorganized

**Figure 5:**
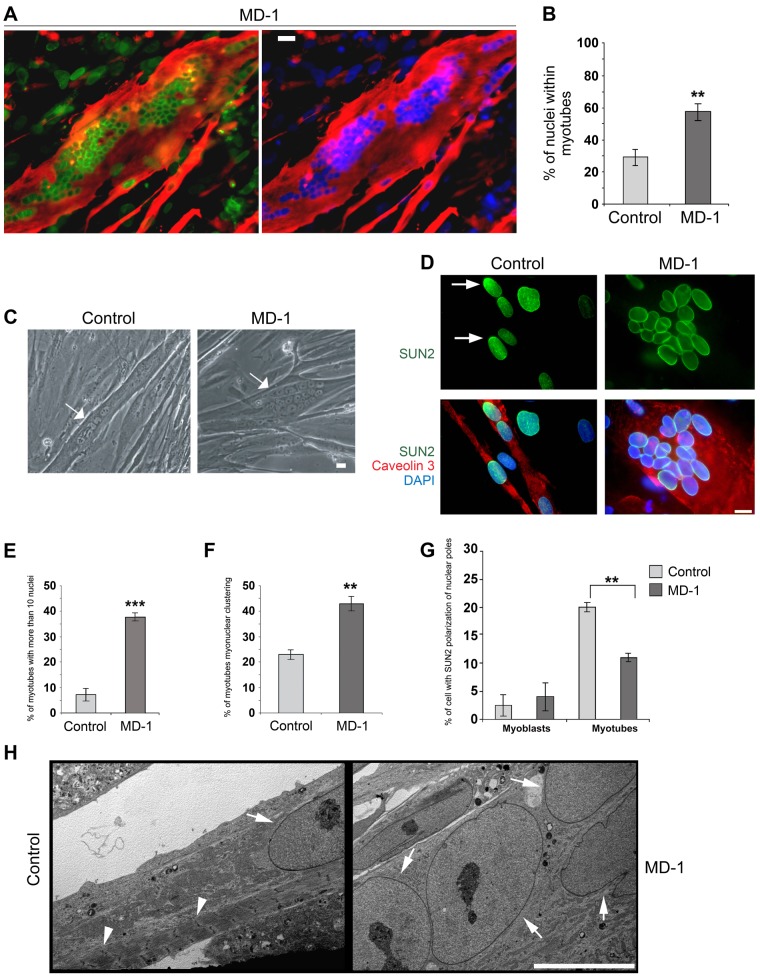
Enhanced rate of differentiation, nuclear misalignment and clustering in MD-1 myotubes. (**A**) Immunofluorescence staining of MD-1 myotubes using SUN1 (green) and desmin antibodies (red). Chromatin was stained with DAPI (blue). Scale bar, 30 µm. (**B**) Graphical representation of the percentage of nuclei within myotubes counted in MD-1 and in five independent control cultures. (**C**) Phase contrast image of living control and MD-1 cultured myotubes (arrows) showing myonuclear clustering in patient cells. Scale bar, 10 µm. (**D**) Immunofluorescence staining of control and MD-1 myotubes with SUN2 (green) and caveolin 3 (red) antibodies. Arrows indicate SUN2 polarization at the nuclear poles in control cells. (**E–F**) Graphical representation of the percentage of myotubes with more than 10 nuclei and the percentage of myotubes with myonuclear clustering in control and MD-1 cultures. Data are presented as mean values ± S.D. of three independent experiments (50 myotubes per sample were counted). (**G**) Graphical representation of the percentage of committed myoblast and myotube nuclei with enrichment of SUN2 staining at the nuclear pole(s). Data are presented as mean values ±S.D. of 3 independent experiments (200 nuclei per sample). (**H**) Transmission electron microscopy analysis of control and MD-1 myotubes (see Materials and Methods for details). Sarcomeric structures are evident in control myotubes (arrowheads), whereas they are absent from MD-1 myotubes showing myonuclear clustering. Arrows indicate myonuclei. Scale bar, 10 µm.

Exogenous expression of EDMD2-associated lamin A/C variants and disruption of endogenous lamin A/C, emerin or the LINC complex all affect myoblast differentiation [60]–[63]. Furthermore, studies in *Drosophila* indicate that the LINC complex is required for correct myonuclear spacing [34]. We therefore determined whether the myoblasts carrying SUN1 variants had defects in their ability to differentiate to form mature, multinuclear myotubes. In order to account for inherent differences in differentiation capacity between cell lines, we compared MD-1 cultures with a total of 5 control cultures independently established from different individuals. SUN1 staining was clearly evident at the nuclear envelope of MD-1 myotubes, as previously observed in myoblasts (Figure 5A). However, we found that the MD-1 myoblasts exhibited an increased rate of differentiation, both in terms of the number of myotubes and the number of nuclei per myotube. There was a striking increase in the percentage of nuclei within myotubes (defined as muscle-specific caveolin3-positive cells containing at least 3 nuclei) in patient cultures (28±6% versus 58±9% in control and MD-1 cultures, respectively; mean ± sem) and a 5-fold increase in myotubes possessing more than 10 nuclei (Figure 5A–E). Strikingly, 45% of these myotubes displayed gross nuclear misalignment and clustering, with up to 30 nuclei per myotube in some instances (Figure 5D,F).

Together with our earlier observation of defective nuclear repositioning in fibroblasts expressing disease-associated variants and in double-mutant EDMD2 fibroblasts, these findings suggested that mutations in *SUN1* and *SUN2* disrupt connections with the cytoskeleton, thereby perturbing nuclear anchorage. Studies have previously shown that SUN1 and SUN2 become concentrated at the poles of the nucleus during human primary myoblast differentiation and that this polarization is linked to correct myonuclear spacing [32]. We therefore examined SUN1 and SUN2 polarization in patient MD-1 myotube nuclei and found that, whilst SUN1 polarization was normal (Figure S5), SUN2 failed to polarize in clustered nuclei (Figure 5D,G). In keeping with earlier observations in myoblasts, we also found that nesprin-2 fluorescence intensity was significantly increased in all patient myotubes (Figure S6). Ultrastructural analysis showed that myonuclear clustering occurred within single MD-1 myotubes (Figure 5H) and further demonstrated that enlarged highly differentiated myotubes with misaligned nuclei were devoid of detectable sarcomeric structures that were visible in controls (Figure 5H, arrowheads).

### Pericentrin recruitment and microtubule nucleation at the NE is defective in MD-1 myotubes

**Figure 6:**
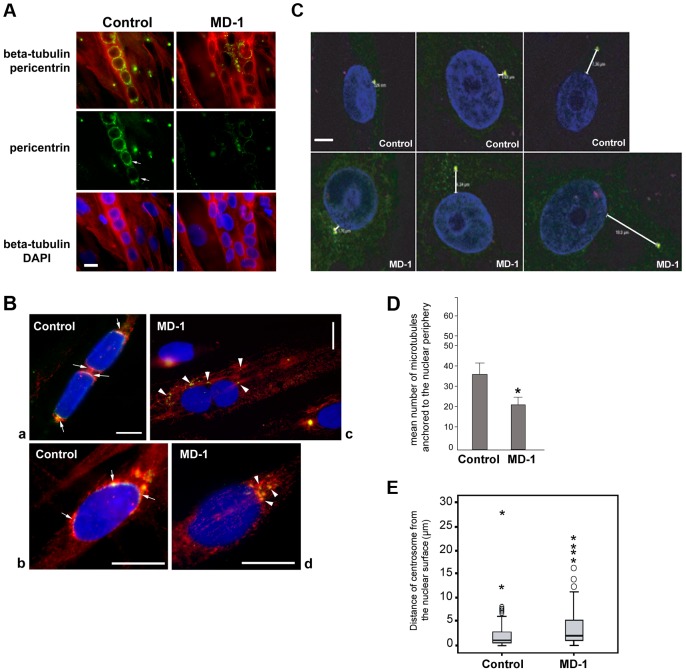
Impaired pericentrin localization and microtubule nucleation at the nuclear envelope in MD-1 myotubes. (**A**) Beta-tubulin (red) and pericentrin (green) double immunofluorescence staining of control and MD-1 myotubes. Chromatin was stained with DAPI (blue). Scale bar, 10 µm. Arrows indicate apparent pericentrin accumulation at the nuclear poles. (**B**) Control and MD-1 myotubes were treated with nocodazole followed by 5 min recovery in culture medium to allow microtubule regrowth. Samples were then fixed and stained as in panel A. Panels a and c show myotubes, whilst panels b and d show committed myoblasts from control and MD-1 cultures, respectively. Arrows in control cells indicate sites of microtubule regrowth at the nuclear poles, co-inciding with pericentrin localization. Arrowheads in patient cells indicate microtubule regrowth from cytoplasmic pericentrin foci. (**C**) Control and MD-1 myoblasts were fixed in methanol and subjected to immunofluorescence analysis using γ-tubulin antibodies to stain the centrosome and DAPI to stain the DNA. Distances between centrosomes and the nuclear periphery (µm) are indicated. Scale bar, 10 µm. (**D**) The number of microtubules nucleating from individual myotube nuclei prepared in B was counted and is presented as the mean ±S.D (n = 50 nuclei per sample). * p<0.05 as calculated using the Student's *t*-test. (**E**) Nucleus-centrosome distance was measured in 100 control and MD-1 myoblasts prepared in C, in two independent experiments, using Leica LAS AF Lite software and analysed using SPSS software. The median values (thick black lines) were 0.96 and 2.43 µm for control and MD-1 cells, respectively. *P* = 0.00012. ○ and * correspond to mild and extreme outliers, respectively.

During myotube formation, the microtubule network is reorganized into a parallel array along the longitudinal axis of the myotube and is nucleated from the nuclear surface, which becomes the primary microtubule organizing centre (MTOC) of the cell [64]. As part of this process, centrosomes undergo partial disassembly and centrosomal proteins, including γ-tubulin, pericentrin and PCM-1, become concentrated at the nuclear periphery [65], [66]. We hypothesized that SUN proteins contribute to centrosomal protein recruitment to the NE and that polarization of SUN proteins at the nuclear poles may promote linear nuclear organization in myotubes. We therefore investigated the recruitment of centrosomal proteins to the NE during myogenesis, using pericentrin as a marker. In control myotubes we found that pericentrin was recruited to the NE and there was a suggestion that it concentrated at the poles of the nuclei, in a similar manner to SUN2 (Figure 6A). In contrast, pericentrin failed to accumulate to any significant degree at the nuclear surface in MD-1 myotubes and was instead found in cytoplasmic foci. These findings support our hypothesis that SUN proteins are involved in the recruitment of pericentrin to the NE in myotubes.

We next investigated whether microtubule nucleation from the NE was disrupted in the patient myotubes by observing microtubule regrowth following nocodazole-induced depolymerization. In control cells, microtubules could be clearly observed emanating from around the nuclear surface after nocodazole wash-out for 30 minutes (Figure S7). In contrast, the microtubule network in patient cells was very disorganized and often did not appear to be attached to the NE, suggesting a defect in microtubule nucleation, anchoring or organization. To investigate this further, we performed a short 5-minute nocodazole wash-out to detect sites of microtubule nucleation. Microtubule asters regrowing from the nuclear envelope were observed in control myotubes (Figure 6B, panel a) and committed myoblasts (Figure 6B, panel b). Microtubule nucleation correlated with sites of pericentrin concentration at the nuclear poles (arrows in Figure 6B). In MD-1 myotubes and committed myoblasts, microtubules were seen to nucleate mainly from multiple sites in the cytoplasm, corresponding with the locations of cytoplasmic pericentrin foci (Figure 6B, arrowheads). Counting fifty myotubes per sample, we could demonstrate that the mean number of microtubules nucleating from myotube nuclei was significantly reduced in MD-1 (Figure 6D).

These data suggested that centrosome attachment to the nucleus may also be disrupted in myoblasts from this patient. We therefore examined nuclear-centrosomal distance in MD-1 myoblasts and indeed observed a 2-fold increase in separation between the nucleus and centrosomes in the patient myoblasts compared to controls (mean distance 4.34 µm versus 2.07 µm, respectively) (Figure 6C,E).

**Figure 7:**
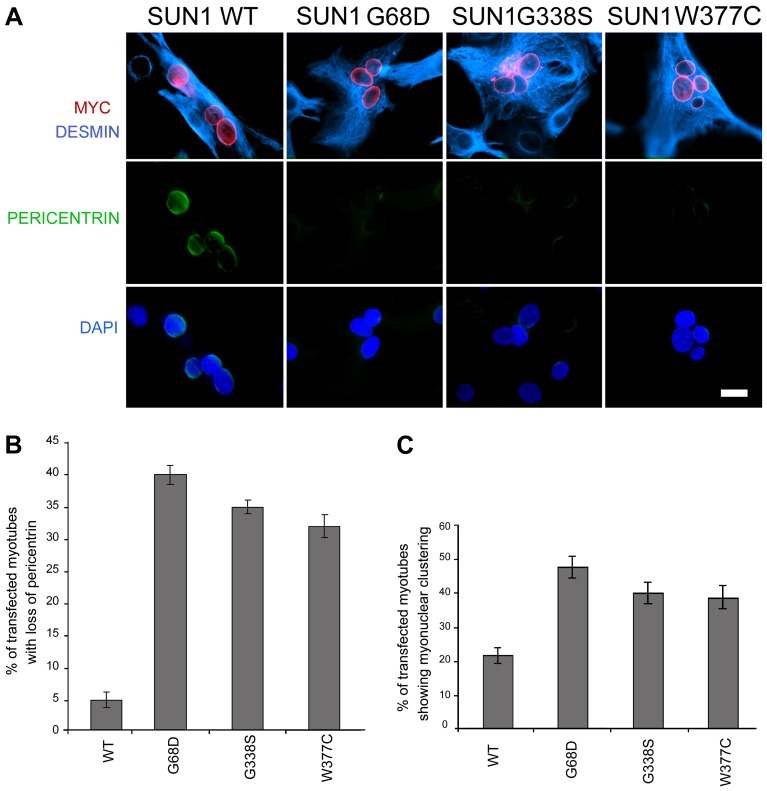
Exogenously expressed SUN1 mutants impair pericentrin recruitment to the nuclear envelope. (**A**) Differentiated C2C12 myotubes transfected with wild-type (WT) SUN1, or the indicated mutants, were labelled with anti-myc (red) and anti-pericentrin antibodies (green). Desmin antibody (violet) was used as a muscle differentiation marker. Nuclei were counterstained using DAPI. Samples were observed using a Nikon laser confocal microscope. Bar, 10 µm. (**B–C**) Transfected myotubes prepared as in A were quantified for the absence of pericentrin staining at the nuclear envelope (B) and myonuclear clustering (C). Thirty myotubes per sample were counted in two independent experiments. Differences for all mutants were statistically significant with respect to wild-type-transfected myotubes (*P*<0.01).

To confirm that loss of pericentrin recruitment to the NE was a direct consequence of SUN1 mutation, we observed pericentrin localization in C2C12 myotubes following transient transfection with myc-SUN1 variants. Pericentrin was absent from the NE of myonuclei expressing SUN1 G68D, G338S and W377C variants, but exhibited clear nuclear rim staining in myonuclei expressing WT SUN1 (Figure 7 A–B). Thus, all 3 mutants tested acted in a dominant manner in C2C12 cells to displace pericentrin from the NE.

Finally, to directly link the SUN1 mutants to the nuclear clustering phenotype observed myotubes of patient MD-1, we assessed the degree of nuclear clustering in myotubes expressing WT SUN1 and the G68D, G338S and W377C variants. Whilst 22% of myotubes expressing WT SUN1 displayed myonuclear clustering, this value was increased by 2-fold in the cultures expressing each of the 3 SUN1 variants (Figure 7C). These findings confirm that the *SUN1* p.G68D and p.G338S mutations are the primary cause of the failure to recruit pericentrin to the NE and the defective nuclear positioning in myotubes from patient MD-1 and further indicate that this is likely to be common to muscle from patients carrying other *SUN1* mutations, including p.W377C.

In summary, our data demonstrate that muscle disease-associated alterations in SUN proteins result in loss of nuclear connectivity to the cytoskeleton. In myotubes, *SUN1* mutations disrupt connections with centrosomal components and the microtubule network, in particular impairing microtubule organization and nucleation from the NE. This in turn is likely to lead to impaired myonuclear positioning in multinuclear myotubes, which we propose may be an important contributor to muscle dysfunction.

## Discussion

We have identified a total of 11 *SUN1* and 7 *SUN2* rare, non-synonymous variants in our cohort of EDMD and related myopathy patients. Ten of these variants, identified in nine unrelated families, had putative pathogenic effects as deduced from genetic and functional analyses.

### Multiple modes of inheritance of *SUN1* and *SUN2* mutations

Our data add to an increasingly complex picture concerning the genetics of EDMD and related myopathies, where multiple genes, either alone or in combination, can cause or modify the disease phenotype. The *SUN1* and *SUN2* variants appear to be inherited in highly variable manners, with or without the presence of a mutation in a second gene. In 2 families (families 1 and 2), *SUN1* or *SUN2* variants were inherited from each of the unaffected parents of the index patients, strongly supporting an autosomal recessive mode of inheritance in those families. One sporadic case carried heterozygous mutations in both the *SUN1* and *SUN2* genes, suggesting that mutations in the 2 genes could have additive effects, as has been observed in *Sun1/Sun2* knockout mice [30]. In other instances, the index case carried only one *SUN1* or *SUN2* variant and often represented a sporadic case, suggestive of either a dominant *de novo* mutation or presence of a concurrent mutation in another, as yet unidentified, gene.

We also identified *SUN1* or *SUN2* variants in individuals from 4 families harbouring known *LMNA* or *EMD* mutations. In all cases, the *SUN1/SUN2* mutation alone did not cause disease in other family members. However, disease severity was significantly increased in the individuals carrying both mutations compared to family members, or unrelated individuals, carrying only the *LMNA* or *EMD* mutation. Furthermore, their phenotype was on the severe end of the spectrum, as defined by Yates et al. [50]. These findings suggest that some *SUN1/SUN2* variants act as modifiers to increase disease severity. There has been much speculation as to the existence of modifier genes in EDMD due to high variability in disease phenotype between affected individuals within families [37], [38], [41], [47] and there is now some evidence to support this. In a family with X-linked EDMD caused by an *EMD* p.Y105X mutation, disease severity was increased in one individual due to a second mutation present in the *LMNA* gene [42]. Similarly, an individual with severe disease and carrying a *LMNA* p.R644C mutation was found to carry a second mutation in the gene encoding desmin [42].

In our cohort, for some cases (such as patient MD-3) the increased severity was expressed as clinically more severe muscular dystrophy [39]. In other cases, the additional presence of a *SUN1/SUN2* mutation was associated with more severe cardiac disease. For example, patient MD-5 from family 5, carrying both *LMNA* p.R453W and *SUN1* p.W377C mutations, developed cardiac disturbances at age 25 and died from heart failure at age 34, which is much earlier than is typical for EDMD patients [50]. Furthermore, *LMNA* p.R453W is a relatively common mutation that has been reported in at least 15 individuals with EDMD and is usually associated with mild disease [8], [48], [67]–[70]. Thus, it is likely that the *SUN1* mutation carried by patient MD-5 contributed to their increased disease severity. We also identified *SUN1* p.W377C in combination with *SUN2* p.E438D in a sporadic case, supporting the idea that a mutation in a second gene is required for disease causation in this instance. Further support for a modifying role for the p.W377C mutation came from the ability of this mutant to worsen nuclear dysmorphology when expressed in *LMNA* R401C patient fibroblasts.

Individuals MD-6 and MD-7 (carrying a *SUN2* variant in addition to *LMNA* T528K and R99P mutations, respectively) also had more severe disease than is typical for EDMD, with early onset at age 1 and 4 years, respectively, and unusually early requirement for heart transplantation. In each of these sporadic cases, the *LMNA* mutation arose *de novo* and so no comparisons can be made with family members, however, their clinical phenotype is consistent with the suggestion that the *SUN2* variants contribute to increased disease severity. Whilst our genetic and cell-based data strongly support a modifying role for *SUN* mutations in some patients, studies involving larger patient cohorts will be necessary to prove this conclusively.

For 8 of the rare, non-synonymous variants identified in our cohort, there was a lack of compelling evidence of their disease association. However, given the complex interplay between mutations in different genes, more investigation is required before entirely ruling out their involvement. In particular, *SUN1* p.V846I was found in an isolated sporadic case and thus no co-segregation analysis could be performed to support its disease association. Yet this is a mutation of highly conserved residue (Figure S3) that lies within the SUN domain that is involved in nesprin binding. Thus it will be important in the future to utilize functional studies to investigate the impact of such mutations on LINC complex interactions.

Despite our findings increasing the number of known EDMD-associated genes to 8, still almost 50% of patients in our cohort have no identified mutations in any of these genes. Most of these patients represent sporadic cases, with no family history of disease, making mutation screening difficult. Furthermore, since 6 of the known genes each account for only a small percentage of cases, it is likely that there are multiple genes remaining to be identified. Proteins associated with the LINC complex are clearly very strong candidates and there are several such proteins that should be examined as a priority, including Samp1 [71], [72].

### Muscular dystrophy-associated *SUN1* and *SUN2* variants disrupt nuclear-cytoskeleton connection and nuclear positioning

Through the various nesprin isoforms expressed at the ONM, the LINC complex mediates attachment to all three cytoskeletal filament networks [17], [73]. In this study, we have demonstrated, in several different systems, that muscular dystrophy-associated mutations in SUN1 or SUN2 impair nuclear coupling to the both actin and microtubule networks and disrupt nuclear movement and positioning.

In mouse NIH3T3 fibroblasts, five out of the six SUN1 and SUN2 variants that we examined inhibited rearward movement of the nucleus, which has previously been shown to be achieved through LINC complex attachment to actin cables closely associated with the nuclear surface [53]. Our data strongly indicate that at least five of the variants identified in our patient cohort have a negative functional impact upon nuclear-cytoskeletal connection via the LINC complex, which is likely to be a major contributor to muscle disease pathophysiology. One of the variants examined, *SUN2* p.M50T, did not impair rearward nuclear movement, suggesting that this variant is not disease-causing and this is entirely possible given the complex genetics in the individual carrying this mutation (patient MD-2). In agreement with our findings, EDMD-associated lamin A variants were recently shown to cause a similar defect in nuclear movement in NIH3T3 cells [52] and disrupted nuclear-cytoskeletal coupling [74].

**Figure 8:**
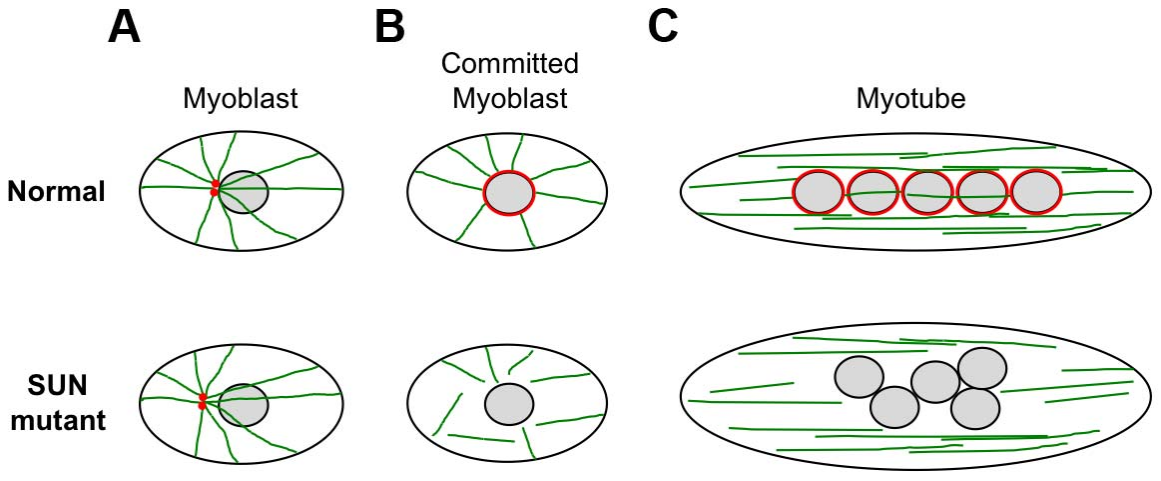
Schematic model of nuclear positioning and microtubule connections during differentiation of normal and SUN1/2 mutant myoblasts. (**A**) In myoblasts, the close positioning of centrosomes (red) to the outer nuclear surface is disrupted by SUN1/2 mutants, which is likely to be accompanied by impaired microtubule (green) association with the NE. (**B**) Upon commitment to differentiation in normal myoblasts, pericentrin and other centrosomal proteins redistribute from the centrosome to the nuclear surface, which becomes the major site of microtubule nucleation. In mutant committed myoblasts, pericentrin fails to associate with the NE and there is impairment of microtubule nucleation from the nuclear surface. (**C**) After cell fusion to form myotubes, the microtubules reorganize into overlapping parallel arrays along the long axis of the cell. The myonuclei become positioned evenly along the length of the cell in a microtubule-dependent manner with the involvement of dynein and kinesin motor proteins. In mutant myotubes, nuclei are clumped in a disorganized fashion and we propose that this is due to an inability to interact with the microtubule network.

We also observed defects in nuclear positioning in differentiating myotubes derived from patient MD-1, carrying compound heterozygous *SUN1* p.G68D/p.G338S mutations, which is consistent with recent findings that proper SUN1 and SUN2 recruitment to the NE is required for myonuclear spacing [32]. In strong support for a direct role of SUN proteins, nuclear positioning in skeletal muscle is disrupted in double Sun1/Sun2 knockout mice or in mice with targeted disruptions of nesprin-1/nesprin-2 and this leads to clustering similar to that observed in our patient myotubes [30], [31], [75]. Thus, the phenotype we observed in patient MD-1 is consistent with a defect in the LINC complex. Several recent studies have shown that myonuclear position is controlled by nuclear attachment to the microtubule network and that this is mediated by the LINC complex [34], [35], [76]–[78]. At the onset of myoblast differentiation, proteins involved in microtubule nucleation redistribute from the centrosome to the NE. Our observations of impaired pericentrin recruitment and microtubule nucleation/organization at the NE in the patient myotubes therefore support a model whereby mutations in SUN proteins impair nuclear-microtubule connection and prevent correct positioning of myonuclei (Figure 8). It is also well established in other systems that unanchored nuclei float freely in the cytoplasm and tend to clump together, as observed in MD-1 myotubes [34], [77], [79].

It is currently not clear how the mutant SUN proteins, which are located at the INM, mediate disruption of microtubule attachment to the NE. Studies have indicated that nesprins are important for microtubule association with the NE, through their interaction with microtubule motor proteins [77], [80]. However, we did not obtain any evidence that the central SUN1-nesprin-2 LINC complex interaction was perturbed in MD-1 myoblasts, suggesting that the defect may lie elsewhere. Instead, SUN1 interaction with emerin was disrupted and, consistent with this, both emerin mRNA and protein levels were reduced in myoblasts from patient MD-1. Impairment of SUN1/SUN2 interaction with emerin has also been observed in cases of EDMD1 due to mutations in emerin itself [44]. Furthermore, EDMD has been associated with defects in emerin interaction with lamin A/C and nesprins [18], [19]. Interestingly, emerin has been shown to partially localize at the ONM, where it may contribute to centrosomal attachment to the NE and, in agreement with our findings, others have observed increased centrosomal separation from the nucleus in EDMD1 cells [81], [82]. Thus, dysregulation of emerin may play a role in disease causation.

### Contribution of SUN mutations to muscle disease pathophysiology

To date, most studies have focused on the role of the nuclear lamina and LINC complex in cellular resistance to mechanical strain in support of the “structural” hypothesis of laminopathy disease causation. There is now strong evidence to indicate that defects in these structural networks make a significant contribution to the pathophysiology of EDMD and related disorders [29]. Given our observations of defective interaction networks in SUN-mutated cells and uncoupling of nuclear-cytoskeletal connections, it is therefore likely that the variants we have discovered in SUN1 and SUN2 also impact upon cell mechanics. Nuclear dysmorphology is a common feature of laminopathy cells and, whilst the exact cause and effect of this phenomenon is not understood, it is likely to reflect changes in the organization of the nuclear lamina and its interactions with the nuclear envelope [83], together with increased susceptibility to mechanical deformation. The exacerbation of nuclear dysmorphology, induced by SUN1 W377C expression in *LMNA* R401C fibroblasts, again highlights a role for SUN proteins in NE organization and integrity.

Our findings in patient MD-1 myotubes further indicate that defects in nuclear positioning may play a significant role in disease pathogenesis, particularly since a link has also been made between loss of myonuclear anchoring and impaired muscle function [34], [35]. Myonuclear positioning defects have been observed at the myotendinous junctions of *Lmna* ^H22P/H22P^ and *Lmna* knock-out mice, and in EDMD patients with mutations in *LMNA* [33], [84]. However, to our knowledge, ours is the first observation of such a pronounced myonuclear mispositioning phenotype in humans. It will be important, in future studies, to demonstrate directly that uncoupling of the nucleus from the microtubule network through *SUN* mutation does lead to muscle disease *in vivo* with physiological expression levels of SUN mutants.

In summary, our data clearly implicate defects in pericentrin recruitment, microtubule nucleation/organization and nuclear-cytoskeletal attachment in NE-associated muscular dystrophy pathogenesis and are in agreement with the bulk of results showing SUN1/SUN2 involvement in nuclear positioning and cell migration. It remains to be determined precisely how centrosomal components are recruited to the nuclear envelope in differentiating myotubes and how defects in this process result in misalignment of myonuclei in muscular dystrophy.

## Materials and Methods

### Ethics statement

This study involved the use of human DNA samples and myoblasts derived from muscle biopsies. These were obtained following informed consent using protocols and consent forms approved by the Ethics Committee of Ernst-Moritz-Arndt University, Greifswald.

### Patients and controls

EDMD patients for this study were selected based on the results of a routine diagnostic mutational analysis of *EMD*, *LMNA*, *FHL1*, *SYNE1* and *SYNE2*. 175 pseudo-anonymized patients negative for mutations in these genes and 70 patients known to carry mutations in the genes encoding the LINC components emerin, lamin A/C and nesprin 1 or 2 alpha and beta were tested for mutations in *SUN1* and *SUN2*. The clinical features of these unrelated, predominantly Caucasian index cases were within the diagnostic criteria for EDMD [50] despite the variable clinical expression.

### Mutation analysis

Primer pairs for all the coding exons and flanking intronic sequences of *SUN1* (*UNC84A*, ENSG00000164828; see Fig. S1) and *SUN2* (*UNC84B*, ENSG00000100242, ENST00000405510) were designed using Primer-Blast (http://www.ncbi.nlm.nih.gov/tools/primer-blast/index.cgi; Table S2). To standardize the sequencing reaction, all primers were tagged with an M13-tail (forward: 5′-GTAAAACGACGGCCAGT-3′ reverse: 5′-CAGGAAACAGCTATGAC-3′). Amplifications were performed in 25 µl volumes using Amplikon-Taq Polymerase (Biomol) under the following thermal conditions: initial denaturation at 94° for 5 min followed by 35 cycles of denaturation (94°C for 15 sec), annealing at the appropriate temperature for 15 sec (see Table S2) and elongation (72°C for 1 min). A final elongation (72°C for 7 min) preceded a 4°C cooling step

Direct Sanger sequencing was used to analyse PCR products. Excess dNTPs and primers were removed using ExoSAP-IT (Affymetrix). Sequencing reactions were performed using ABI BigDye Terminator v3.1 Cycle Sequencing Kit with addition of 5% DMSO to the reaction mix. M13-oligonucleotides were used as sequencing primers. The reactions were analysed on a 3130xl GA DNA Sequencer (Applied Biosystems) according to the manufacturer's instructions. All DNA variations identified were validated using a second independent DNA sample.

### Analysis of the frequency of DNA variations

Unique and rare sequence variations were tested for their frequency in 400 alleles of a Caucasian reference population. Additionally, sequence variations found in a patient of Turkish origin were tested in 138 alleles of a Turkish reference population. Co-segregation of DNA variations with the disease was analysed in patient families if available. For estimating the frequency of DNA variations found, restriction digestion and high resolution melting (HRM) were performed using patient DNA as positive control. Restriction enzymes cutting specifically at the DNA variation were selected using NEB-cutter (http://tools.neb.com/NEBcutter2/). HRM products amplified with LightCycler 480 High Resolution Melting Master (Roche) were analysed on a LightCycler 480 II (Roche) according to the manufacturer's instructions. Samples showing abnormal signals were examined by restriction endonuclease digestion or direct sequencing. The frequency of changes found in patients of different origin was estimated from online accessible genome sequencing data (Table S1).

### Real-time PCR

RNA was extracted from patient MD-1 and control myoblasts using TRIzol (Invitrogen) according to the manufacturer's instructions. Real-time PCR was performed using a RealTime ready custom panel and LightCycler 480 Probes Master (Roche), with primers as described in Supplementary Material Table S3, and evaluated on a LightCycler 480 II (Roche), according to the manufacturer's instructions. Values for each gene were normalised to both actin and GAPDH.

### Plasmid constructs and site-directed mutagenesis

For plasmid constructs, the pCMVTag3B vector (Stratagene) was used to fuse a myc tag to the N-terminus of SUN1. The 916 amino acid version of the SUN1 cDNA, lacking the ATG start codon, was generated by PCR amplification in two stages. First, codons 2–362 were amplified using primers 5′-CACAGAATTCGATTTTTCTCGGCTTCACAT-3′ and 5′-CACAGTCGACCTATCCGATCCTGCGCAAGATCTGC-3′ with IMAGE clone 40148216 as template and inserted into pCMVTag3B via the *Eco*RI and *Sal*I sites. This introduced a *Bgl*II site via a silent mutation at codon 356–358. Codons 352–916 were then amplified using 5′-TTACTTCTTGCTGCAGATCTTGCGCAGGATCGG-3′ and 5′-GAGAGTCGACTCACTTGACAGGTTCGCCATG-3′ from an oligo dT-primed reverse transcription of U2OS cell mRNA and cloned into the *Bgl*II-*Sal*I sites of the initial construct. EDMD-associated mutations were introduced using the QuikChange II site-directed mutagenesis kit (Stratagene), according to the manufacturer's instructions.

### Antibodies

Anti-human SUN1 2383 and anti-human SUN2 2853 antibodies have been described previously [44]. Anti-SUN1 Atlas antibody (HPA008346) was obtained from Sigma prestige antibodies. Anti-nesprin-2 (N2N3) antibody was kind gift from Q. Zhang (King's College London) and has been described previously [16]. Anti-nesprin-2G has been reported previously [53]. Anti-nesprin-2 monoclonal antibody (IQ562) was purchased from Immuquest. Monoclonal anti-emerin antibody was a kind gift from G. Morris (Center for Inherited Neuromuscular Disease, Oswestry, UK). Anti-lamin A/C (sc-6215) and GFP antibodies were purchased from Santa Cruz Biotechnologies. Anti- GAPDH (MAM374) was obtained from Millipore. Anti-α-tubulin (T9026), anti-β-actin (A5441), anti-γ-tubulin (T6557), anti-myc and anti-desmin antibodies were purchased from Sigma. Anti-caveolin 3 monoclonal antibody (610420) was purchased from Transduction Laboratories and anti-desmin polyclonal antibody (MONX10657) was purchased from Monosan. Anti-pericentrin polyclonal antibody (Ab4448) was obtained from Abcam.

### Cell culture and transfection

Myoblasts from patient MD-1 and controls were routinely cultured in high-glucose DMEM supplemented with 20% foetal bovine serum plus antibiotics penicillin, streptomycin and amphotericin B, at 37°C and 5% CO2, and were used between passages 3 and 7. Myoblasts at confluence were allowed to differentiate into myotubes in the same culture medium for 8–15 days, replacing the medium every 5 days. HeLa cells were cultured in DMEM supplemented with 10% FBS and antibiotics. For emerin co-immunoprecipitation experiments, HEK293 cells were transfected with the appropriate pCMVTag3-SUN1 constructs together with GFP-emerin [85] using Fugene 6 (Promega), according to the manufacturer's instructions. pCMVTag3-SUN1 constructs were transfected into C2C12 mouse myotubes using the Amaxa Nucleofector (Lonza), according to the manufacturer's instructions. Cultures were fixed 24 hours after transfection and processed for immunofluorescence analysis.

### Centrosome reorientation and nuclear movement assay

NIH3T3 fibroblasts were cultured in 10% calf serum in DMEM (Gibco) as previously described [86]. Following serum starvation for two days, confluent monolayers were “wounded” by removing a strip of cells and nuclei of cells at the edge of the wound were microinjected with the appropriate myc-tagged SUN1 or SUN2 DNA plasmids. After expression for 2 hr, cells were stimulated with 10 µM LPA for 2 hr, fixed in 4% paraformaldehyde, extracted with Triton X-100 and stained with antibodies to tyrosinated α-tubulin (rat monoclonal antibody at 1/40 of culture supernatant), myc (mouse monoclonal antibody from clone 9E10, Roche) and DAPI (Sigma) followed by appropriate secondary antibodies. Stained samples were observed with a Nikon TE300 microscope using a 40× Plan Apo N.A. = 1.0 or 60× Plan-Apo N.A. = 1.4 objective and filter cubes optimized for DAPI, fluorescein/GFP, and rhodamine. Images were acquired with CoolSNAP HQ camera (Photometrics) driven by Metamorph software (MDS Analytical Technologies) and further processed in Image J. Centrosomes were considered oriented if they were localized in the pie-shaped sector between the nuclear membrane the leading edge scored, as described [86], [87]. Random orientation is ∼33% by this measure. Nuclear and centrosome position relative to the cell centroid were determined as described [88]. Data were plotted as % of the cell radius to normalize for differences in cell size.

### Cell extracts, immunoprecipitation, and immunoblotting

To prepare total cell extracts for immunoblotting, cells were scraped into cold 1×phosphate-buffered saline (PBS), pelleted by centrifuging at 200×*g* for 5 min and then pellets were resuspended in lysis buffer (10 mM HEPES [pH 7.4], 5 mM EDTA, 50 mM NaCl, 1% Triton X-100, 0.1% SDS) supplemented with 1 mM PMSF and protease inhibitor cocktail (Roche) and an equal volume of Laemmli buffer was then added. For human myoblast immunoprecipitations, cells were grown on 10 cm dishes and then immunoprecipitated as described previously (Haque et al., 2006) using 2 µg of SUN1 2383 antibody. 5% of the initial lysate was retained for immunoblot analysis. All samples were boiled in an equal volume of 2×Laemmli buffer, resolved on 6% or 7.5% or 10% polyacrylamide gels, followed by semidry transfer onto nitrocellulose membrane. Membranes were probed using the appropriate primary antibodies and dilutions: hSUN1 ATLAS (1∶400), hSUN2 2853 (1∶500), lamin A/C (1∶2000), emerin (1∶1500), nesprin-2 N2N3 (1∶1500), α-tubulin (1∶ 10,000), β-actin (1∶20,000), GAPDH (1∶ 10,000). Primary antibodies were detected using horseradish peroxidase-conjugated secondary antibodies (Sigma), and visualization was performed using ECL reagents (Geneflow).

### Indirect immunofluorescence microscopy

Myoblasts and myotubes grown on glass coverslips were fixed in methanol at −20°C and processed for indirect immunofluorescence microscopy as previously described (Haque et al., 2006). For SUN1 staining, cells were instead fixed in 4% paraformaldehyde and permeabilized with 0.5% Triton X-100 at room temperature for 5 min. Cells were washed in PBS and incubated with antibodies diluted in PBS–3% bovine serum albumin, using hSUN1 2383 (1∶150), hSUN2 2853 (1∶100), lamin A/C (1∶400), emerin (1∶500), nesprin-2G (1∶300), γ-tubulin (1∶500) pericentrin (1∶50), caveolin 3 (1∶30) and desmin (1∶100) antibodies. Secondary antibodies were goat anti-rabbit AlexaFluor 488, donkey anti-mouse AlexaFluor 594 and donkey anti-goat AlexaFluor 594 (Molecular Probes Inc.). DNA was stained with 50 µg/ml 4′,6-diamidino-2-phenylindole (DAPI; Sigma). Coverslips were mounted in 80% glycerol–3% *n*-propyl gallate (in PBS) or ProLong gold antifading reagent (Invitrogen). Fluorescence microscopy was performed with a Nikon TE300 inverted microscope with an ORCA-R^2^ charge-couple device camera (Hamamatsu) and Volocity software (PerkinElmer). Where required fluorescence microscopy was also performed with Leica TCS SP5 confocal laser scanning microscope and Leica LAS AF software. Images were processed with Adobe Photoshop (Adobe Systems). Quantification of fluorescence intensity was performed using an Olympus Scan∧R microscope with a 20× objective. Approximately 1000 nuclei from 3 independent experiments were randomly selected by their DAPI signal, and the intensity of SUN1, SUN2, emerin, lamin A/C and nesprin-2 was measured within the DAPI-stained region.

### Electron microscopy

Myotubes (at passage 2–3) from patient and age-matched controls were fixed in 2.5% glutaraldehyde-0.1 M cacodylate buffer pH 7.4 for 3 h at 4°C. After post-fixation with 1% osmium tetroxide (OsO4) in cacodylate buffer for 2 h, samples were dehydrated in an ethanol series, infiltrated with propylene oxide and embedded in Epon resin. Ultrathin sections (60 nm thick) were stained with uranyl acetate and lead citrate (10 min each) and were observed at 0° tilt angle with a Geol Jem 1011 transmission electron microscope, operated at 100 kV. At least 30 myoblasts/myotubes per sample were observed.

### Statistical analysis

In all cases, statistical analysis was performed using a Student's *t*-test to compare differences in values obtained for patient/mutant versus control samples.

## Supporting Information

Figure S1 Transcript variant of *SUN1* used in this study. (A) The 23-exon *SUN1* isoform used for our investigations contained exons 4 to 26 of ENST00000456758. The start codon used is the same used in isoform ENST00000405266. (B) The resulting isoform encodes 916 residues and corresponds to the full length mouse isoform of SUN1 that predominates in most tissues [89]. Alternating exons are indicated in black and blue. Residues spanning splice sites are indicated in red.
(TIF)

Figure S2 Pedigrees of MD families with index patients carrying heterozygous *SUN1* or *SUN2* variants that do not co-segregate with disease. Index cases are indicated by arrows. There was no evidence of increased disease severity in the index cases carrying the *SUN1* variants.
(PDF)

Figure S3 Evolutionary conservation of SUN1 and SUN2 mutated residues. All rare, non-synonymous variants identified in SUN1 and SUN2 are shown. Those for which there is strong genetic and/or functional evidence of disease-association are indicated in red. The mutated residues and their equivalents in other species are highlighted in beige.
(PDF)

Figure S4 SUN1 mRNA levels are not altered in MD-1 myoblasts. Expression level of the indicated genes was assessed by quantitative real-time PCR using total RNA isolated from control and MD-1 myoblasts. Values are expressed relative to two control genes, *ACTB* and *GAPDH*, and show the average of 2 independent experiments performed in duplicate ±S.E. Significant *P*-values are as follows: *SUN2 P* = 0.019, *LMNA P* = 0.009, *SYNE1 P* = 0.0016, *SYNE2 P* = 0.01.
(PDF)

Figure S5 SUN1 can polarize in MD-1 myotubes. SUN1 (green) and caveolin (red) immunofluorescence staining in control and patient MD-1 myotubes, along with DAPI (blue) staining of DNA. Arrowheads indicate nuclei in which SUN1 is enriched at the poles.
(TIF)

Figure S6 Nesprin-2 expression is elevated in patient MD-1 myotubes. (A) Nesprin-2 staining in myotubes from control and patient MD-1. Immunofluorescence labeling was performed with nesprin-2 monoclonal (red) and desmin (green) antibodies. Desmin was used as a muscle cell marker. (B) Nesprin-2 fluorescence intensity was measured using the NIS software analysis system and 50 myotubes per sample were analysed. Data are presented as mean value ±S.D. Significant *P*-value for patient MD-1 was 0.042. Scale bar, 10 µm.
(TIF)

Figure S7 The number of microtubules nucleating from the nuclear envelope is reduced in MD-1 myotubes. Beta-tubulin (red) and pericentrin (green) double immunofluorescence staining in untreated control and MD-1 myotubes, or following nocodazole treatment and 30 min recovery in culture medium. Chromatin was stained with DAPI (blue). Scale bar, 10 µm. Higher magnification (3×) of nuclear envelopes in nocodazole-treated cells is shown on the right of each picture.
(TIF)

Table S1 Single nucleotide changes found in coding regions of *SUN1* and *SUN2* and their frequencies in sequenced genome databases. Rare, non-synonymous variants are highlighted in bold, with blue shading. *Patient MD-1 was of Turkish origin, therefore 150 alleles from ethnically matched controls were also screened for mutations p.G68D and p.G338S.
(PDF)

Table S2 Primer sequences and annealing temperatures for genomic amplification of *SUN1* and *SUN2* exons.
(PDF)

Table S3 Primers used for real-time PCR.
(PDF)
