# Supplementary figures and images for: Muscular Dystrophy-Associated SUN1 and SUN2 Variants Disrupt Nuclear-Cytoskeletal Connections and Myonuclear Organization
Source: PLoS Genet. 2014 Sep 11;10(9):e1004605. doi: 10.1371/journal.pgen.1004605 (PMC4161305; doi:10.1371/journal.pgen.1004605)

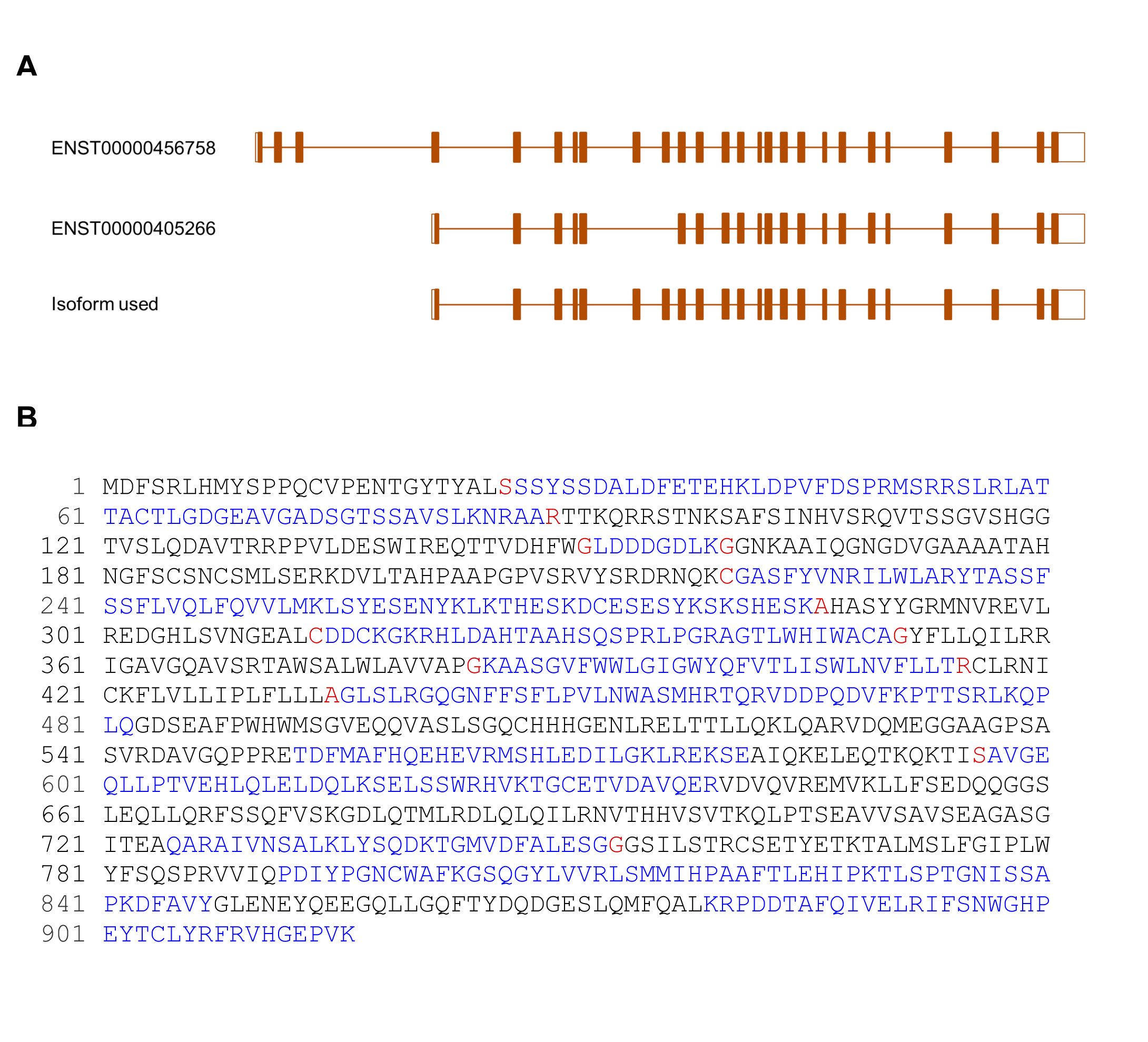

Supplement: Figure S1 — Transcript variant of SUN1 used in this study. (A) The 23-exon SUN1 isoform used for our investigations contained exons 4 to 26 of ENST00000456758. The start codon used is the same used in isoform ENST00000405266. (B) The resulting isoform encodes 916 residues and corresponds to the full length mouse isoform of SUN1 that predominates in most tissues [89]. Alternating exons are indicated in black and blue. Residues spanning splice sites are indicated in red. (TIF) [file pgen.1004605.s001.tif]

**Figure S2**

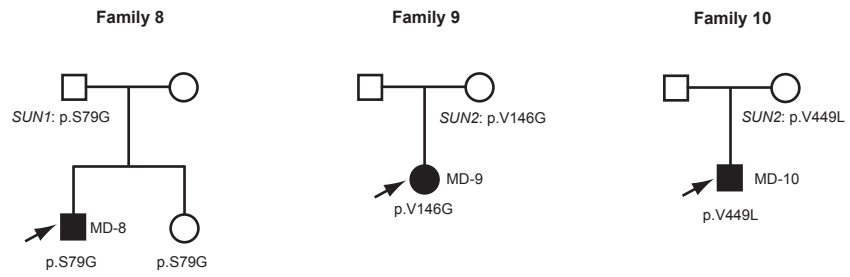

Supplement: Figure S2 — Pedigrees of MD families with index patients carrying heterozygous SUN1 or SUN2 variants that do not co-segregate with disease. Index cases are indicated by arrows. There was no evidence of increased disease severity in the index cases carrying the SUN1 variants. (PDF) [file pgen.1004605.s002.pdf]

Figure S4

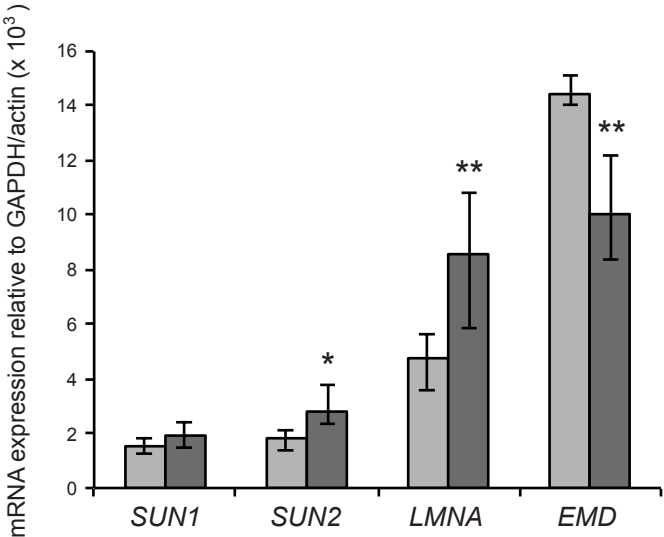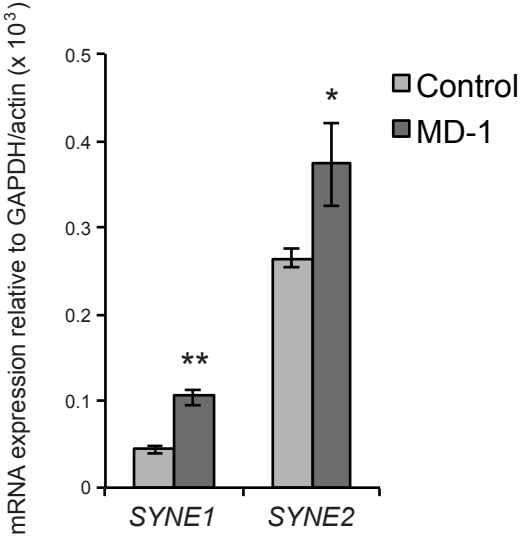

Supplement: Figure S4 — SUN1 mRNA levels are not altered in MD-1 myoblasts. Expression level of the indicated genes was assessed by quantitative real-time PCR using total RNA isolated from control and MD-1 myoblasts. Values are expressed relative to two control genes, ACTB and GAPDH, and show the average of 2 independent experiments performed in duplicate ±S.E. Significant P-values are as follows: SUN2 P = 0.019, LMNA P = 0.009, SYNE1 P = 0.0016, SYNE2 P = 0.01. (PDF) [file pgen.1004605.s004.pdf]

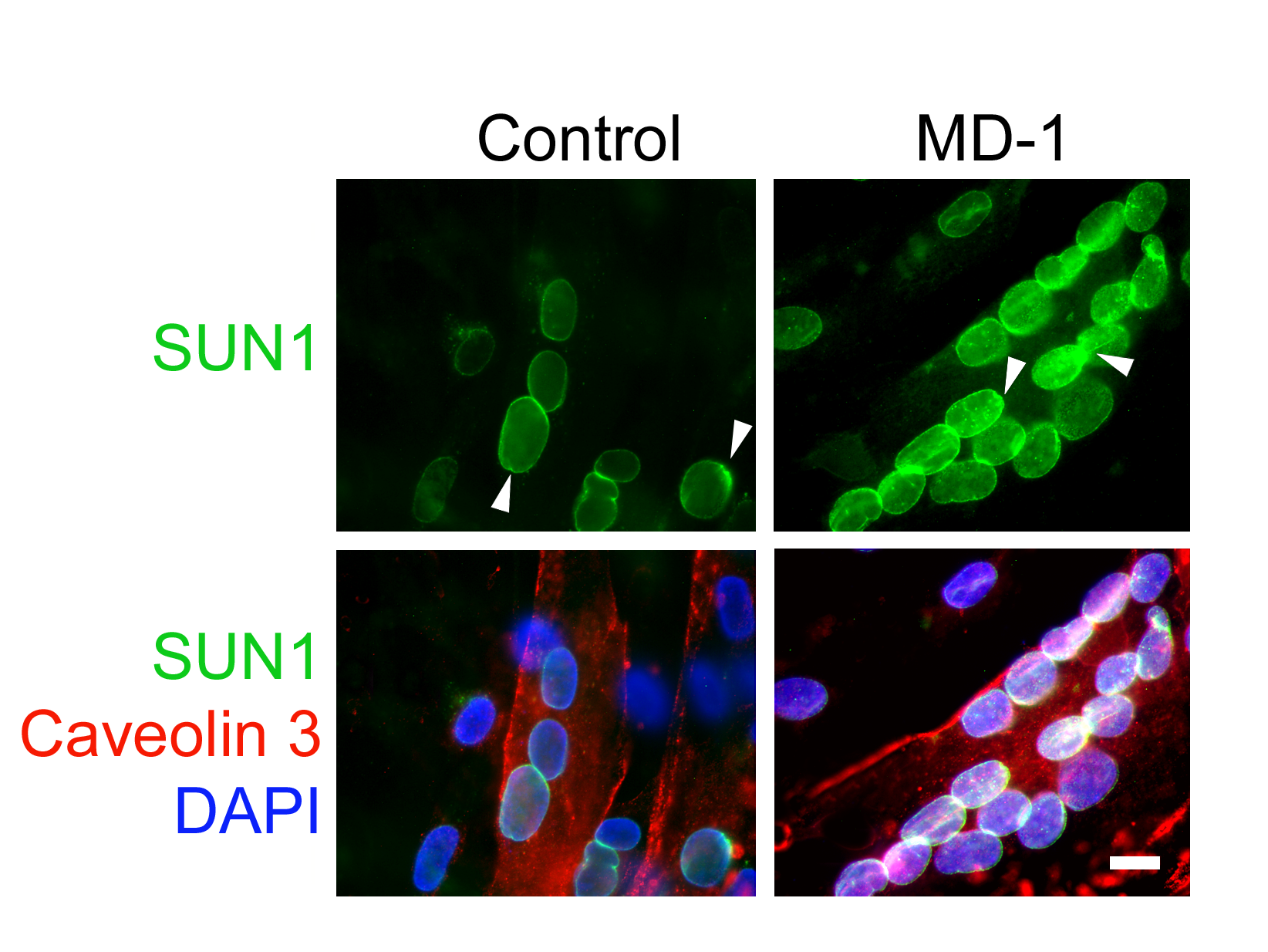

Supplement: Figure S5 — SUN1 can polarize in MD-1 myotubes. SUN1 (green) and caveolin (red) immunofluorescence staining in control and patient MD-1 myotubes, along with DAPI (blue) staining of DNA. Arrowheads indicate nuclei in which SUN1 is enriched at the poles. (TIF) [file pgen.1004605.s005.tif]

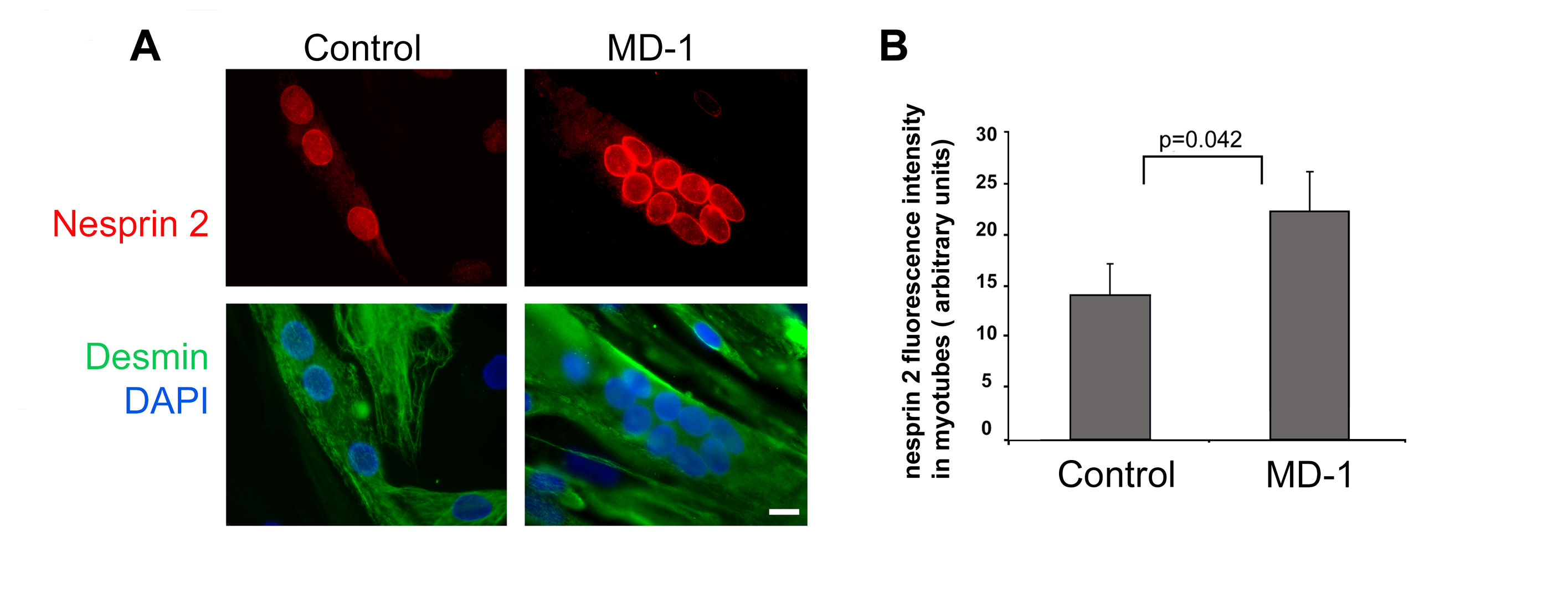

Supplement: Figure S6 — Nesprin-2 expression is elevated in patient MD-1 myotubes. (A) Nesprin-2 staining in myotubes from control and patient MD-1. Immunofluorescence labeling was performed with nesprin-2 monoclonal (red) and desmin (green) antibodies. Desmin was used as a muscle cell marker. (B) Nesprin-2 fluorescence intensity was measured using the NIS software analysis system and 50 myotubes per sample were analysed. Data are presented as mean value ±S.D. Significant P-value for patient MD-1 was 0.042. Scale bar, 10 µm. (TIF) [file pgen.1004605.s006.tif]

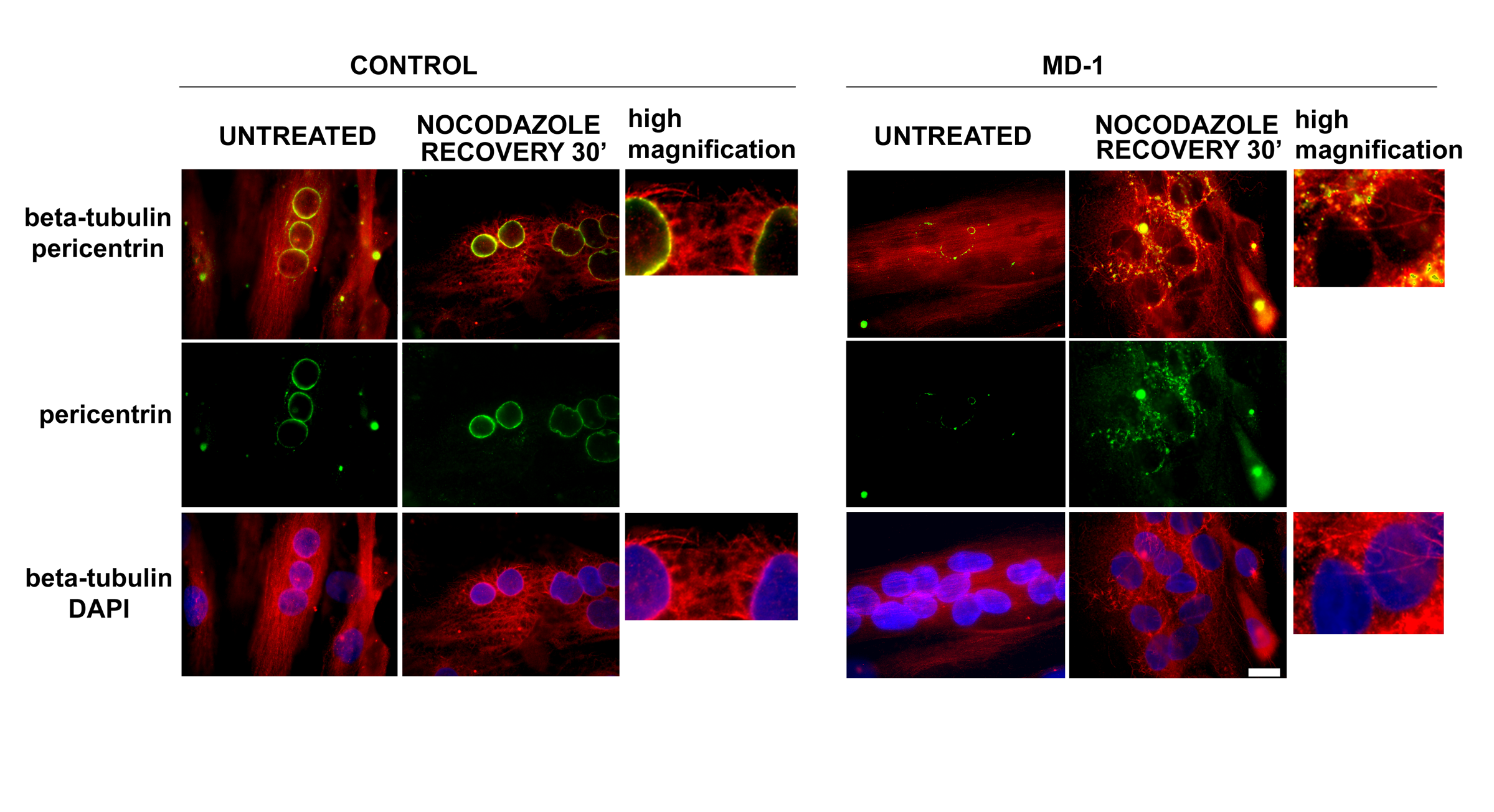

Supplement: Figure S7 — The number of microtubules nucleating from the nuclear envelope is reduced in MD-1 myotubes. Beta-tubulin (red) and pericentrin (green) double immunofluorescence staining in untreated control and MD-1 myotubes, or following nocodazole treatment and 30 min recovery in culture medium. Chromatin was stained with DAPI (blue). Scale bar, 10 µm. Higher magnification (3×) of nuclear envelopes in nocodazole-treated cells is shown on the right of each picture. (TIF) [file pgen.1004605.s007.tif]
