# Supplementary material for: Muscular Dystrophy-Associated SUN1 and SUN2 Variants Disrupt Nuclear-Cytoskeletal Connections and Myonuclear Organization
Source: PLoS Genet. 2014 Sep 11;10(9):e1004605. doi: 10.1371/journal.pgen.1004605 (PMC4161305; doi:10.1371/journal.pgen.1004605)
Supplement: Figure S3 — Evolutionary conservation of SUN1 and SUN2 mutated residues. All rare, non-synonymous variants identified in SUN1 and SUN2 are shown. Those for which there is strong genetic and/or functional evidence of disease-association are indicated in red. The mutated residues and their equivalents in other species are highlighted in beige. (PDF) [file pgen.1004605.s003.pdf]

Figure S3

SUN1	G68D	G76A	S79G	R94H	V197M	A203V	A325T	G338S	W377C	A718V	V846I
Homo sapiens	LGDGEAV	ADSGTSS	GTSSAVS	TKQRRST	RKDVLTA	AHPAAPG	HLDAHTA	RLPGRAG	SALWLAV	EAGASGI	DFAVYGL
Pongo abelii	LGDGEAV	ADSGTSS	GTSSAVS	AKQHRSA	RKDVLTA	AHPVVPG	HLDAHTA	RPPGRAG	SALWLAV	EAGASGI	DFAVYGL
Mus musculus	SGDSQAI	SHISTSR	STSRATP	VKQRRSA	RTDALTA	AHSAIHG	HLEIHTA	QPHRVAG	SVLWLAV	QAGISGI	DFAVYGL
Cavia porcellus	SGDSQAM	THSCASS	CASSTAS	VKQRRST	RTDMLTA	AAPAAHG	HLETHTA	KLHRVVG	SVLWLAI	EAGISGI	DFAVYGL
Monodelphis domestica	SDDGQVE	THSYTRN	YTRNTAY	SKQHRNT	RKDVLTA	AYSTSHV	HLETYTT	RSKRVAR	SFLWLAI	SLGISGI	DFSVYGL
Taeniopygia guttata	SDDGQSD	DSIHDSS	HDSSYAG	VKQRRSM	RKEVLTA	AYSASPV	LLYAPRL	RSDGMAC	SLLWLAI	NAGISGI	NFAVYGL
Gallus gallus	KPDSARN	DLLHDSS	HDSSYAG	VKQRKSI	RKEVLTA	AYSASSV	QLEIHTT	RAKRVAR	SLLWLAI	NAGISGI	KFSVYGL
Xenopus tropicalis	ADNSATS	ICAGTLS	GTLSKCS	MQQHNSS	RNDALTA	ALSASYS	HHEMRTT	WARGVTG	SFLWLAI	TGAIDGI	DFAVYGL
Danio rerio	YDNSFTE	EVAGNHS	GNHSVGS	TTTTSSS	GNEKETS	SYSVPYS	HMEMNTE	RMLTVLW	SILWFAV	TAEAAGM	RFSVYGL

SUN2	M50T	A56P	V146G	V378I	E438D	V449L	R620C
Homo sapiens	SSNMKRL	LSPAPQL	YSDVDQQ	KKIVRAS	VAEEVGL	IQAVRDD	ARIRPTA
Pongo abelii	SSNMKRL	LSPAPQL	YSDADQQ	KKIVRAS	VADEVGL	FQAVRDD	ARIRPTA
Mus musculus	SSNMKHL	LSPAPQL	YADIEQH	KKIVQAS	VADEVGL	IQAARAD	ARIRPTA
Cavia porcellus	SSNMKHL	LSPVPQL	YADIEQH	KKIVQAS	VEDEVGL	IQAVREE	ARIRPTA
Monodelphis domestica	SGSMKRL	LSPAPHL	NLGMDQP	KKIIQAF	LCPLVGS	IGHATHA	ALIRPTA
Taeniopygia guttata	SSSTKRL	LSPTPST	HYYSGQS	GKMAQAS	MGKHVKG	LKTVRAD	GIIRPTA
Gallus gallus	KSINKQS	LSRASDT	GWLVSQK	RKLSAES	VDAQVKE	KMMLFGD	MKIYPTA
Xenopus tropicalis	RRKVTTL	LKHAPTP	MADVQQQ	QKMHNMF	LSQQVET	IQGVRDG	SPIHPTA
Danio rerio	SRNSSRA	ASSVSLI	HYEQPIK	LMRDIEL	LHSTQDL	VDALEAH	YPVAITH
