# Supplementary material for: Muscular Dystrophy-Associated SUN1 and SUN2 Variants Disrupt Nuclear-Cytoskeletal Connections and Myonuclear Organization
Source: PLoS Genet. 2014 Sep 11;10(9):e1004605. doi: 10.1371/journal.pgen.1004605 (PMC4161305; doi:10.1371/journal.pgen.1004605)
Supplement: Table S1 — Single nucleotide changes found in coding regions of SUN1 and SUN2 and their frequencies in sequenced genome databases. Rare, non-synonymous variants are highlighted in bold, with blue shading. *Patient MD-1 was of Turkish origin, therefore 150 alleles from ethnically matched controls were also screened for mutations p.G68D and p.G338S. (PDF) [file pgen.1004605.s008.pdf]

Table S1

Gene	DNA variation	Amino acid exchange	Frequency in all patients (n=490 alleles)	Frequency in reference population (n=400 alleles)	rs-number	NHLBI-ESP:ESP_Cohort _Populations[1]	1000GENOMES: phase_1_ALL[2]
SUN1	c.114 G>A	p. T38T	3	N/D	rs76073621	---	87/2097
SUN1	**c.203 G>A**	**p. G68D**	1	---*	rs188935423	---	4/2180
SUN1	**c.227 G>C**	**p. G76A**	1	N/D	rs149038179	---	5/2179
SUN1	**c.235 A>G**	**p. S79G**	1	---		---	---
SUN1	**c.281 G>A**	**p. R94H**	1	N/D	rs112598200	---	1/2183
SUN1	c.352 C>T	p. H118Y	203	N/D	rs6461378	---	1153/1031
SUN1	c.366 C>T	p. V122V	6	N/D	rs41273066	---	13/2171
SUN1	c.543 C>T	p. N181N	1	N/D		---	---
SUN1	**c.589 G>A**	**p. V197M**	1	---	rs116520953	---	7/2177
SUN1	c.606 C>T	p. P202P	21	N/D	rs113652875	---	54/2130
SUN1	**c.608 C>T**	**p. A203V**	1	N/D	rs144929525	---	4/2180
SUN1	c.869 A>G	p. Y290C	28	N/D	rs74742245	---	358/1826
SUN1	**c.973 G>A**	**p. A325T**	2	N/D	rs139112134	---	7/2177
SUN1	**c.1013 G>A**	**p. G338S**	1	1*		---	---
SUN1	**c.1131 G>T**	**p. W377C**	2	---	rs142011077	---	5/2179
SUN1	c.1815 A>G	p. T605T	160	N/D	rs10950789	---	968/1216
SUN1	c.1819 G>A	p. E607K	76	N/D	rs59910530	---	359/1825
SUN1	**c.2153 C>T**	**p. A718V**	1	---	rs114701323	---	1/2183
SUN1	c.2436 G>A	p. R812R	6	N/D	rs61744747	---	94/2090
SUN1	**c.2536 G>A**	**p. V846I**	1	1		---	---
SUN1	c.2592 G>A	p. T864T	1	N/D		---	---
SUN2	c.97 A>G	p. T33A	2	N/D	rs2072799	5/4545	103/2081
SUN2	**c.149 T>C**	**p. M50T**	1	---		---	---
SUN2	**c.166 G>C**	**p. A56P**	1	N/D	rs137966643	2/4550	---
SUN2	c.266 T>G	p. L89R	52	N/D	rs35496634	351/4197	128/2056
SUN2	**c.437 T>G**	**p.V146G**	1	---		---	---
SUN2	c.657 G>A	p. P219P	1	N/D	rs143617240	5/4547	---
SUN2	c.1042 C>T	p. R348C	12	N/D	rs138708	59/4491	84/2100
SUN2	**c.1132 G>A**	**p. V378I**	4	3	rs139004902	26/4522	12/2172
SUN2	**c.1314 A>T**	**p. E438D**	1	2		---	---
SUN2	**c.1345 G>C**	**p. V449L**	1	---		---	---
SUN2	c.1824 A>G	p. Q608Q	151	N/D	rs1062687	1708/2734	860/1324
SUN2	**c.1858 C>T**	**p. R620C**	1	---	rs141013997	3/4433	---
SUN2	c.2011 G>A	p. G671S	49	N/D	rs2072797	363/4185	248/1936

---, not detected

[1] Data from ENSEMBL: 1000GENOMES:phase_1_ALL: http://www.ncbi.nlm.nih.gov/SNP/snp_viewTable.cgi?pop=14318

[2] Data from ENSEMBL: NHLBI-ESP:ESP_CohortPopulations http://www.1000genomes.org/faq/which-populations-are-part-your-study
