# Supplementary material for: Muscular Dystrophy-Associated SUN1 and SUN2 Variants Disrupt Nuclear-Cytoskeletal Connections and Myonuclear Organization
Source: PLoS Genet. 2014 Sep 11;10(9):e1004605. doi: 10.1371/journal.pgen.1004605 (PMC4161305; doi:10.1371/journal.pgen.1004605)
Supplement: Table S2 — Primer sequences and annealing temperatures for genomic amplification of SUN1 and SUN2 exons. (PDF) [file pgen.1004605.s009.pdf]

## Table S2

| Gene/Exon | Orientation | Primer sequence (5'-3') | Amplicon size (bp) | Annealing temperature (°C) |
|---|---|---|---|---|
| *SUN1* E1 | F | TTTCCTGCCCGTTAAAACAC | 227 | 62 |
| | R | TGGAGTTCCTGTTAAGGTCCA | | |
| *SUN1* E2 | F | CATTTTGTTTCAGAATGGGG | 312 | 61 |
| | R | TGGAAGCTTGGTTTTAACTGG | | |
| *SUN1* E3 | F | TGCTGTTTATTCCCAGATTAAGAG | 323 | 62 |
| | R | ACTGTAGGCGGGACAATCTG | | |
| *SUN1* E4/5 | F | GACCAGTGCCATAATGCTG | 536 | 64 |
| | R | TCCAAGACAAATAGTGCAGACAC | | |
| *SUN1* E6 | F | GGGCTCTTCCCCAAGTGATT | 452 | 64 |
| | R | CAGCACCAAGACCACAGGAG | | |
| *SUN1* E7 | F | CTAGCCTTGCACATCCTCT | 320 | 64 |
| | R | CAACAGCCCGTCGTCATCAG | | |
| *SUN1* E8 | F | GAATCCGCCACACTCACTG | 190 | 64 |
| | R | GTGACAGGGCGAGATGAGAC | | |
| *SUN1* E9 | F | GTCACTGCAGAGGCTGGTC | 218 | 64 |
| | R | AGGTGCTTAGCTGCCAGAAG | | |
| *SUN1* E10 | F | CATTTAGGAGCTTGGTTTTCC | 194 | 62 |
| | R | CTTAAAGCACCACGCTGAC | | |
| *SUN1* E11/12 | F | GATTGGTGAACCCTGACTTTC | 440 | 62 |
| | R | GTGCAGTGCCAGCTGAGAG | | |
| *SUN1* E13 | F | TGCTTGTTTGACTACCTGGTTTC | 304 | 64 |
| | R | CAGAAGCGCGTTGGTGTC | | |
| *SUN1* E14 | F | GTAGAAACGCCTTGGCCTG | 245 | 62 |
| | R | GACCAAAGGGCTTTAAAATATTAG | | |
| *SUN1* E15 | F | GGGTATTCCAGATAAATCAATGG | 206 | 62 |
| | R | CGCTCCACATAACCACACAC | | |
| *SUN1* E16 | F | CCCCTCAGCCTGTGTGTAGTG | 283 | 61 |
| | R | AAACCTAAGTCATCAATTCAAGTTTTC | | |
| *SUN1* E17 | F | TGGTCCTGCATTATTGGAGGGGTT | 551 | 58 |
| | R | CACACCCCAGCCAGACCACT | | |
| *SUN1* E18 | F | TGGTGTTTGGTCTTCCGTC | 183 | 64 |
| | R | AAGTGCACACCCCTGCTAAC | | |
| *SUN1* E19 | F | TGGTGGAACTGGACACTGAG | 214 | 64 |
| | R | CACTAACCGAGTCCATGCTG | | |
| *SUN1* E20 | F | TCCTCTTCCTGCTGCTAAGTG | 320 | 64 |
| | R | CAGTGCGCTCACGTCACT | | |
| *SUN1* E21 | F | TAGTGCTGGCTGTGGAAGG | 240 | 64 |
| | R | CCTGCTGTCACGAGAAGTTG | | |
| *SUN1* E22 | F | CAGACGTCATATTTGGGGAAG | 594 | 62 |
| | R | TATCTGCCCTCCAAAAGTCC | | |
| *SUN2* E1 | F | CTAGAGAGGAGCTGGGGACA | 203 | 63 |
| | R | AGGTTGCAACAAGAAGGCAG | | |
| *SUN2* E2 | F | GCCAGCATTGGGAGAGTCAG | 410 | 52 |
| | R | GCAGAGAGGTGCTCTGAGGA | | |
| *SUN2* E3 | F | CTCACAGTGTCCCCTGCAGT | 363 | 59 |
| | R | CACATGCTGCCCTCTCTCCA | | |
| *SUN2* E4 | F | CATTCCTTACTGCACACCGG | 344 | 55 |
| | R | GGTCCTACAGTCTCTCTGCA | | |
| *SUN2* E5 | F | CAGGACAGAGGCATACTGGC | 361 | 50 |
| | R | GGGAAGCTACTCCGTGATCG | | |
| *SUN2* E6 | F | GCTCTAGTTCAGCACCCAGG | 262 | 59 |
| | R | CTGCCTGCCAAAGGTCACCA | | |

| | | | | |
|---|---|---|---|---|
| *SUN2* E7 | F | CTGGCAGGGATCAGGTAGCC | 240 | 50 |
| | R | GGGAGCTACCACCCTCAGAG | | |
| *SUN2* E8 | F | GCATGGCCTTATGCTGGAAC | 494 | 55 |
| | R | GCAGAGCTGTCTGATCCCAA | | |
| *SUN2* E9 | F | GCCACTTCCTAGTCATGAGG | 213 | 50 |
| | R | GGTGAACATGTTGGATGGGG | | |
| *SUN2* E10 | F | GACGCTAACAGACAGCAGAT | 251 | 49 |
| | R | CCTACCATCTGCTTGGCAAG | | |
| *SUN2* E11 | F | GTTCACAATGAGTGCTGGGC | 406 | 54 |
| | R | CTTGGGCATAACAGAGGCTG | | |
| *SUN2* E12 | F | CCTGCTATCCAGCTAAGCAC | 419 | 52 |
| | R | CACTTCCATCCTGGAACCTG | | |
| *SUN2* E13 | F | CACAGTGCAAGCCTAGAAGG | 276 | 55 |
| | R | CACCAACCTGGTAGATGCCA | | |
| *SUN2* E14 | F | CTCGGTTAGGGTTAAAGGCT | 349 | 55 |
| | R | GAAAGTCATGTCAGGACAGG | | |
| *SUN2* E15 | F | CATCTGGGGTCAGCAAGAGC | 346 | 61 |
| | R | CCAAATCCACTCCCCTCCCT | | |
| *SUN2* E16 | F | GAAGTGACTTGTCATAGGCC | 351 | 55 |
| | R | CATCTTGAGCTGTGGGAAAG | | |
| *SUN2* E17 | F | GGAGCTTTTGAAGCCTGGTT | 407 | 52 |
| | R | CCCGTCCTTTTCATCTGCAT | | |
