# Supplementary material for: Muscular Dystrophy-Associated SUN1 and SUN2 Variants Disrupt Nuclear-Cytoskeletal Connections and Myonuclear Organization
Source: PLoS Genet. 2014 Sep 11;10(9):e1004605. doi: 10.1371/journal.pgen.1004605 (PMC4161305; doi:10.1371/journal.pgen.1004605)
Supplement: Table S3 — Primers used for real-time PCR. (PDF) [file pgen.1004605.s010.pdf]

Table S3

Gene	Primers
SUN1	5'-TCAGCTTCGGTCAGAGACG-3'
	5'-TGGTGAAAGGCCATAAAGTCA-3'
SUN2	5'-AAACTGCTGCTCGCATCC-3'
	5'-GAGTCTTGCTGATGCTCTGCT-3'
LMNA	5'-GCTCAGTGACTGTGGTTGAGG-3'
	5'-AGCGCAGGTTGTACTCAGC-3'
EMD	5'-GACTTCATTCCCAGATGCTGA-3'
	5'-TACATGGGGCGTTCCCTA-3'
SYNE1	5'-GCAAATCAGCCAAATTAAGGTC-3'
	5'-TGAGCAAAAGACTGGGCTTC-3'
SYNE2	5'-CAAATCTGTTTTGGATCAAGATGA-3'
	5'-GCCTTTGGCAATAAGATGCT-3'
ACTB	5'-GGCCAGGTCATCACCATT-3'
	5'-GGATGCCACAGGACTCCAT-3'
GAPDH	5'-CTCTGCTCCTCCTGTTCGAC-3'
	5'-ACGACCAAATCCGTTGACTC-3'
